# Costimulation blockade in combination with IL-2 permits regulatory T cell sparing immunomodulation that inhibits autoimmunity

**DOI:** 10.1038/s41467-022-34477-1

**Published:** 2022-11-09

**Authors:** Chun Jing Wang, Lina Petersone, Natalie M. Edner, Frank Heuts, Vitalijs Ovcinnikovs, Elisavet Ntavli, Alexandros Kogimtzis, Astrid Fabri, Yassin Elfaki, Luke P. Houghton, Ralf J. Hosse, David A. Schubert, Andreas P. Frei, Ellen M. Ross, Lucy S. K. Walker

**Affiliations:** 1 Institute of Immunity & Transplantation, Pears Building, University College London Division of Infection & Immunity, London, UK; 2 Roche Innovation Center Zurich, Roche Pharma Research & Early Development (pRED), Schlieren, Switzerland; 3 Roche Innovation Center Basel, Roche Pharma Research & Early Development (pRED), Basel, Switzerland

**Keywords:** Peripheral tolerance, Immunosuppression, Regulatory T cells, Interleukins

## Abstract

Blockade of CD28 costimulation with CTLA-4-Ig/Abatacept is used to dampen effector T cell responses in autoimmune and transplantation settings. However, a significant drawback of this approach is impaired regulatory T cell homeostasis that requires CD28 signaling. Therefore, strategies that restrict the effects of costimulation blockade to effector T cells would be advantageous. Here we probe the relative roles of CD28 and IL-2 in maintaining Treg. We find provision of IL-2 counteracts the regulatory T cell loss induced by costimulation blockade while minimally affecting the conventional T cell compartment. These data suggest that combining costimulation blockade with IL-2 treatment may selectively impair effector T cell responses while maintaining regulatory T cells. Using a mouse model of autoimmune diabetes, we show combined therapy supports regulatory T cell homeostasis and protects from disease. These findings are recapitulated in humanised mice using clinically relevant reagents and provide an exemplar for rational use of a second immunotherapy to offset known limitations of the first.

## Abstract (web summary)

The blockade of CD28 signalling is known to reduce pathological T cell responses in the context of both autoimmunity and transplantation, but has been associated with impairment of the regulatory T cell compartment. Here the authors show combining costimulation blockade with the administration of interleukin 2 selectively impairs the T effector response whilst maintaining the regulatory T cell pool and suggest functional effect in a murine model of autoimmune diabetes.

## Introduction

The CD28/CTLA-4 checkpoint is a critical arbiter of T cell activation and a hub of therapeutic intervention in both cancer and autoimmunity. Antibodies targeting CTLA-4 are used to boost anti-tumor immunity^1^ while soluble CTLA-4 molecules, that outcompete CD28 for access to their shared ligands CD80 and CD86, are used in autoimmune settings^2,3^. Both interventions directly or indirectly impact the Treg compartment. CTLA-4 plays an important role in Treg function in mice^4–6^ and humans^7,8^, and antibodies targeting this molecule compromise Treg suppressive capacity, and potentially even delete Treg^9^. This complements effects of the antibody on the conventional T cell (Tconv) compartment, since immune function is boosted regardless of whether the anti-CTLA-4 antibody binds to Treg or Tconv.

In contrast, in the case of soluble CTLA-4 molecules that block CD28 stimulation by binding to CD80 and CD86, the impact on Treg is therapeutically undesirable. By blocking costimulatory ligands, soluble CTLA-4 molecules inhibit Tconv activation but concomitantly impair Treg homeostasis. Accordingly, CTLA-4-Ig fusion proteins^10,11^ or antibodies to CD80 and CD86^12,13^ decrease the frequency of Treg in mice. Likewise, clinical use of the CTLA-4-Ig molecule Abatacept is associated with reductions in circulating Treg in humans^14–17^ as well as a decline in Treg in the inflamed synovium^18^. Given the powerful immunosuppressive properties of Treg, it is clearly suboptimal to reduce this population whilst aiming to suppress immune activation. Consequently, strategies that restrict the effects of costimulation blockade to the conventional T cell compartment, while sparing Treg, would be highly beneficial.

The detrimental effect of CTLA-4-Ig on Treg homeostasis reflects the importance of CD28 signaling for the maintenance of this population^10,19^. Treg homeostasis is also controlled by IL-2 signaling^20–22^, however the relationship between CD28 and IL-2 in maintaining Treg populations has not been fully explored. In this study, we tested whether provision of low dose IL-2 could prevent the Treg impairment associated with blockade of the CD28 pathway. We show that provision of IL-2 can compensate for costimulation blockade in Treg, but not Tconv, and that combining these interventions elicits robust regulation of autoimmunity in a mouse model of diabetes. IL-2 can also preserve Treg homeostasis after Abatacept treatment in humanised mice, indicating a similar interrelationship between the CD28 and IL-2 pathways in human T cells.

These findings support a combination strategy whereby the detrimental effects of costimulation blockade on Treg are offset by concomitant administration of IL-2 therapy. More broadly, the work serves as an exemplar of a functional complementation approach in which mechanistic understanding of one therapy informs the rationale identification of a companion intervention.

## Results

### Blocking CD28 costimulation impairs Treg homeostasis

**Fig. 1:**
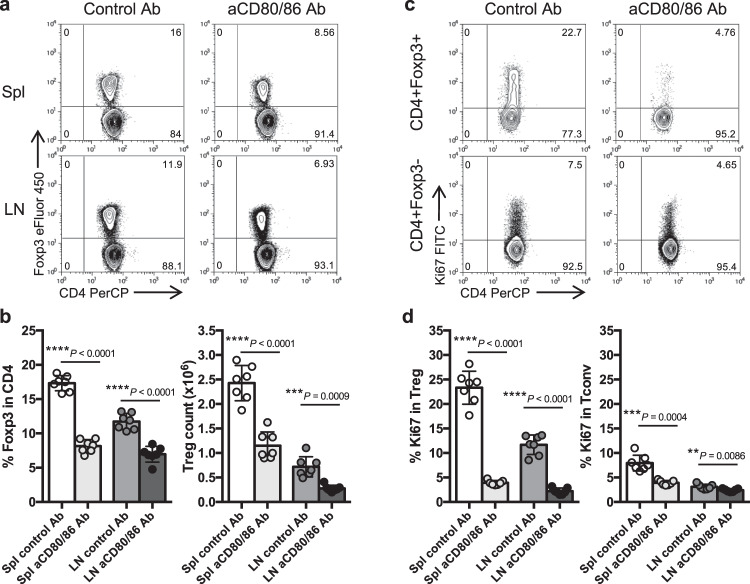
Impact of CD28 costimulation blockade on Treg homeostasis. 6–9 week old BALB/c mice were injected i.p. with anti-CD80/86 Ab or control Ab on d0 and d6. At d8, spleen (Spl) and peripheral lymph node (LN) cells were harvested for analysis. **a** Representative FACS plots show Treg percentage in gated CD4 + cells from either spleen or peripheral lymph nodes **b**. Collated data for Treg percentage (left) and Treg absolute number (right) (*n* = 7 per group). Representative FACS plots from spleen **c** and pooled data **d** illustrate Ki67 expression on Treg (CD4 + Foxp3+) or Tconv (CD4 + Foxp3-) (*n* = 7 per group). Data are collated from 3 independent experiments. Graphs **b** and **d** show mean±s.d.; each dot represents one mouse. **P < 0.01, ***P < 0.001, ****P < 0.0001 (two-tailed *t* test). Source data are provided as a Source Data file.

Mice lacking CD28, or its ligands CD80 and CD86, have decreased proportions of CD4 + Foxp3+ cells, illustrating the importance of the CD28 pathway for Treg homeostasis^10^. Using blocking antibodies to CD80 and CD86 in mice, we confirmed that interrupting CD28 costimulation led to a decrease in the proportion and absolute number of Treg (Fig. 1a, b). Staining with Ki67 revealed that costimulation blockade had a major impact on the proliferation of Treg and a smaller impact on the proliferation of Tconv (Fig. 1c, d). This is consistent with the observation that blockade of CD28 costimulation with Abatacept in the clinic impairs Treg homeostasis in multiple autoimmune settings^14,15,17,18^, including new onset type 1 diabetes^16^, a finding we have recently confirmed^23^.

### IL-2 modulates Treg homeostasis independently of CD28

**Fig. 2:**
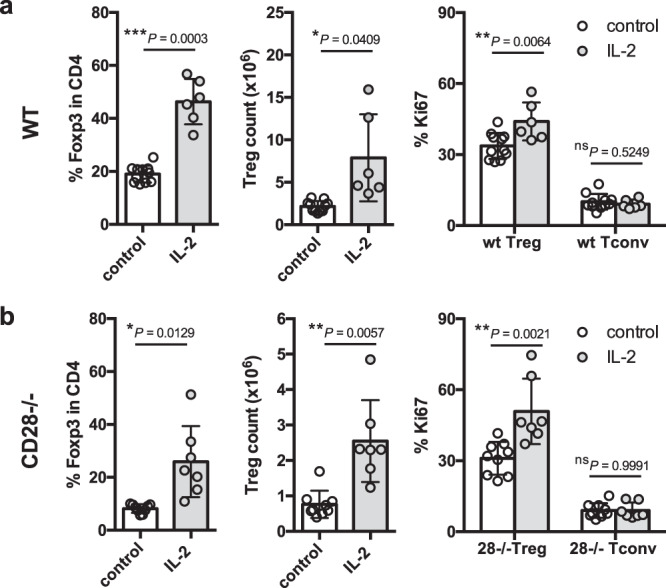
IL-2 promotes Treg homeostasis in CD28-deficient mice. 6–8 week old BALB/c and CD28^−/−^ mice were injected i.p. with IL-2 complex or control on d0, d2, d4, d6 and d7. At d8, spleen cells were harvested for analysis. Graphs show collated data for Treg percentage in CD4 + cells (left), Treg absolute number (middle) and Ki67 expression on Treg or Tconv (right) from treated BALB/c **a**, or CD28^−/−^ **b** mice. Data are presented as mean±s.d.; each dot indicates one mouse. **a** *n* = 11 for control, *n* = 6 for IL-2; (**b**) *n* = 9 for control, *n* = 7 for IL-2. Data are collated from 5 independent experiments. *P < 0.05, **P < 0.01, ***P < 0.001, ns not significant (two-tailed *t* test). Source data are provided as a Source Data file.

Like CD28 costimulation, IL-2 signaling is known to be important for Treg homeostasis^20–22,24,25^, however the relative roles of CD28 and IL-2 in this context are not clear. For example, whether the IL-2 pathway needs to integrate with CD28-derived signals to support Treg maintenance has not been fully established. To address this question, we tested whether IL-2:anti-IL-2 Ab complexes (IL-2c), that have been shown to expand Treg in wildtype mice^26–28^, were capable of performing this function in CD28 deficient mice. We observed that injection of IL-2c increased the percentage, absolute number and proliferation of Treg in wildtype mice (Fig. 2a), and a similar response was seen in CD28-deficient mice (Fig. 2b). Thus, the ability of IL-2 to augment the Treg population did not depend on CD28 signals.

### IL-2 compensates for the absence of CD28 costimulation in Treg

**Fig. 3:**
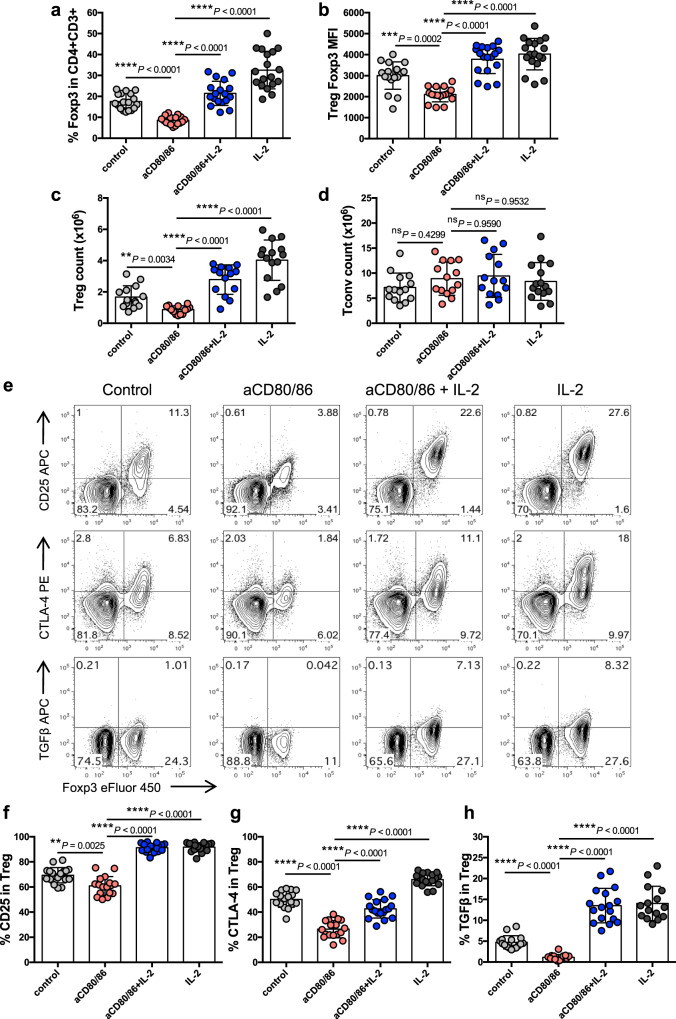
IL-2 can compensate for CD28 blockade in Treg homeostasis. 7–15 week old BALB/c mice were injected i.p. with control Ab, anti-CD80/86 Ab, IL-2 complex or both anti-CD80/86 Ab and IL-2 complex as indicated (anti-CD80/86 Ab was injected on d0 and d6; IL-2 complex was injected on d0, d2, d5 and d7). Spleen cells were analysed at d8. Graphs show collated data for Treg percentage in gated CD4 + CD3 + cells **a**, Treg Foxp3 MFI **b**, Treg absolute number **c**, and Tconv absolute number **d**. Representative FACS plots **e**, and pooled data depict Treg expression of CD25 **f**, CTLA-4 **g**, and TGFβ **h**. Graphs show mean±s.d.; each dot indicates one mouse. **a**, **b**, **f**, **g** *n* = 17 for aCD80/86, *n* = 18 for other groups; **c**, **d** *n* = 15 for IL-2, *n* = 14 for other groups; **h** *n* = 16 for control, *n* = 15 for aCD80/86, *n* = 17 for aCD80/86+IL-2, *n* = 15 for IL-2. Data are collated from 5 independent experiments. **P < 0.01, ***P < 0.001, ****P < 0.0001, ns not significant (ANOVA). Source data are provided as a Source Data file.

The above data raised the possibility that IL-2 could be used to compensate for a lack of CD28 signaling and maintain Treg homeostasis in settings where costimulatory ligands were blocked. To test this idea, wildtype mice were treated with blocking antibodies to CD80 and CD86 with or without co-injection of IL-2c. As before, costimulation blockade markedly reduced the percentage of Treg, however this was restored upon co-injection of IL-2c (Fig. 3a). Likewise, Treg Foxp3 MFI (Fig. 3b) and Treg absolute cell number (Fig. 3c) were decreased by costimulation blockade but these changes were prevented if IL-2c was also provided. In contrast, the effects of IL-2c on Tconv were minimal (Fig. 3d), presumably reflecting the lower expression of CD25 (IL-2Rα) in Tconv compared with Treg.

It has previously been shown that CD25 is downregulated on Treg following injection of anti-CD80/86 Ab^13^, so our finding that IL-2 could still operate effectively in the apparent absence of its high affinity receptor was surprising. Analysis of CD25 expression level confirmed the decrease after costimulation blockade, but revealed that CD25 expression was maintained in animals where IL-2c was co-injected (Fig. 3e, f). Importantly, expression of additional effector molecules on Treg, including CTLA-4 and TGFβ, was also restored when IL-2 was combined with costimulation blockade (Fig. 3e, g, h). Further phenotypic information on Treg from treated animals is provided in Supplementary Fig. 1.

### Regulation of autoimmunity by combining IL-2 and costimulation blockade

These data prompted us to question whether combining IL-2 treatment with costimulation blockade might represent an effective strategy to inhibit autoimmunity. This strategy leverages the immunosuppressive benefits of costimulation blockade without the coincidental loss of Treg. We opted to test this approach in the DO11 × RIPmOVA model of diabetes, in which ovalbumin (OVA)-specific DO11.10 T cells instigate immune-mediated destruction of pancreatic β-cells bearing OVA, since it has been shown that IL-2 complexes alone are ineffective at regulating disease in this strain^29^. Use of this strain therefore allowed us to model a setting in which IL-2 monotherapy was insufficient for disease modulation.

**Fig. 4:**
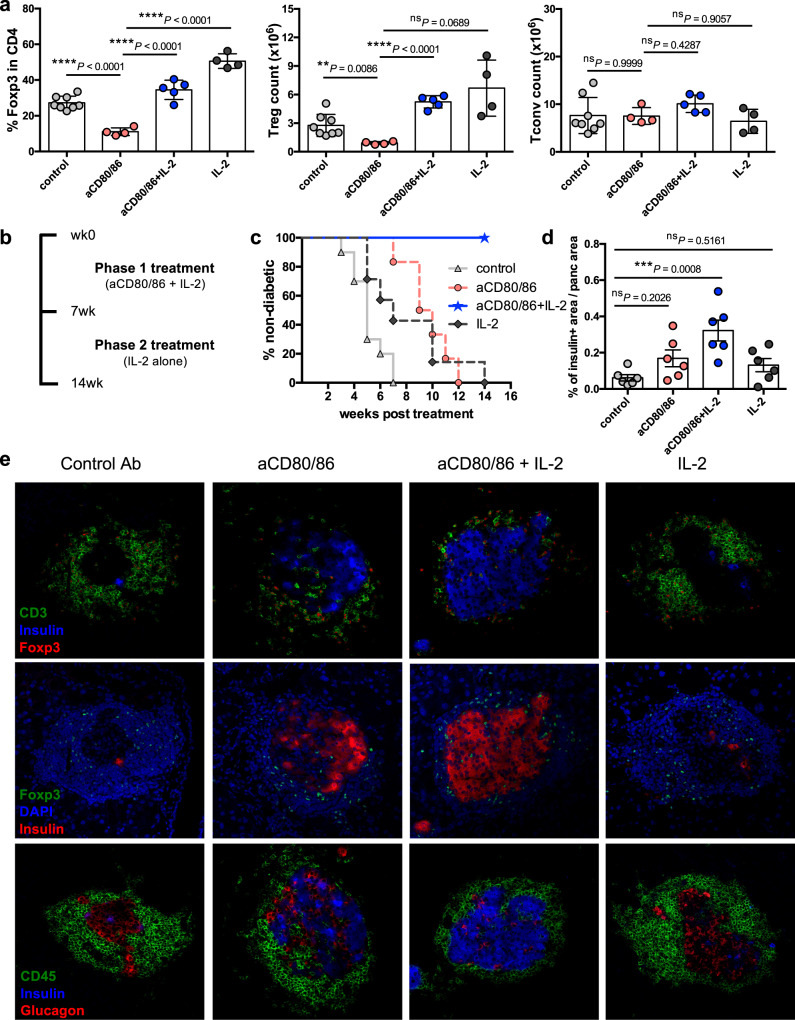
Impact of costimulation blockade and IL-2 on diabetes development in a preventative setting. **a** DO11 × RIPmOVA mice were treated i.p. with control Ab, anti-CD80/86 Ab, IL-2 complex or both anti-CD80/86 Ab and IL-2 complex (two doses of anti-CD80/86 and seven doses of IL-2 complex). Spleen cells were analysed at d8. Graphs show collated data for Treg percentage in gated CD4 + cells (left), Treg absolute number (middle) and Tconv absolute number (right). Data are presented as mean±s.d.; each dot indicates one mouse. *n* = 8 for control, *n* = 4 for aCD80/86, *n* = 5 for aCD80/86+IL-2, *n* = 4 for IL-2. Data are collated from 3 independent experiments. **P < 0.01, ****P < 0.0001, ns = not significant (ANOVA). **b** 2-phase combination treatment protocol. 4–6 week old normoglycaemic DO11 × RIPmOVA mice were treated with anti-CD80/86 Ab plus IL-2 complex for 7 weeks (phase 1). Subsequently, mice were maintained on IL-2 complex alone (phase 2). Control groups received either treatment alone or control Ab (not depicted). **c** Percentage of non-diabetic mice based on blood glucose measurements (*n* = 10 for control, *n* = 6 for anti-CD80/86, *n* = 9 for anti-CD80/86+IL-2, *n* = 7 for IL-2; Data are collated from 3 independent experiments). *P* values were determined by Log-rank test with Bonferroni correction. Values for the anti-CD80/86 Ab group (**P = 0.0017) and anti-CD80/86 Ab plus IL-2 complex group (****P < 0.0001) were significantly different from the control group. **d** At the end of the experiment, frozen pancreas sections from 6 mice for each treatment group were stained for CD4 and insulin by immunohistochemistry and the percentage of pancreas area positive for insulin was calculated for each mouse (*n* = 6 per group, total of 182 islets for control group, 292 islets for anti-CD80/86 group, 424 islets for anti-CD80/86+IL-2 group, 296 islets for IL-2 group). ***P < 0.001, ns not significant (ANOVA). **e** Frozen pancreas sections from mice from each treatment group were stained by immunofluorescence for the indicated markers. Representative confocal microscopy images are shown (20× magnification, 1.75 zoom). Source data are provided as a Source Data file.

We first sought to establish whether short-term combination treatment modulated Treg homeostasis in DO11 × RIPmOVA mice in a similar manner to that seen in wildtype mice; we predicted that IL-2 treatment might be more problematic in autoimmune settings given the upregulation of CD25 on activated T cells. However, similar to what was observed in wildtype mice, the percentage of Treg was decreased by costimulation blockade but this was offset by simultaneous provision of IL-2c (Fig. 4a). IL-2 also restored Treg absolute numbers in the face of costimulation blockade, while absolute numbers of Tconv were not altered (Fig. 4a).

To test the implications for pancreatic islet destruction and blood glucose homeostasis, we adopted a 2-phase treatment protocol in which normoglycaemic DO11 x RIPmOVA mice were treated with anti-CD80/86 under the cover of IL-2c for 7 weeks, followed by withdrawal of costimulation blockade and a treatment period with IL-2c alone (Fig. 4b). Withdrawal of costimulation blockade was intended to re-instate CD28 and particularly CTLA-4-dependent functions in Treg, for example CTLA-4-mediated ligand transendocytosis^30^. We reasoned that an initial period of combination therapy might skew the Treg:Tconv ratio which could subsequently be maintained by IL-2c alone.

Remarkably animals treated with the combined regimen were completely protected from diabetes while those given either treatment alone all developed diabetes (Fig. 4c). As expected, mice treated with anti-CD80/86 Ab alone showed a delay in diabetes development but rapidly developed disease upon treatment cessation (Fig. 4c); analysis of blood samples confirmed a loss of Treg in these mice (Supplementary Fig. 2a). This is consistent with data from clinical trials of costimulation blockade in human type 1 diabetes, in which disease was modestly delayed (9.6 months)^31^ and Treg homeostasis was impaired^16^. Protection from diabetes in recipients of combination therapy was associated with clear preservation of the Treg compartment (Supplementary Fig. 2a). One advantage of the TCR transgenic model is that clonotypic antibody staining can be used to identify Treg that are specific for pancreatic islet antigen. Interestingly, although costimulation blockade impaired the homeostasis of both clonotype-positive and clonotype-negative Treg, addition of IL-2 appeared to selectively preserve the former population (Supplementary Fig. 2b), suggesting that combination therapy may favour Treg undergoing antigen-driven stimulation during an ongoing autoimmune response. The islet-specific conventional T cells in the same blood samples showed decreased expression of activation markers after costimulation blockade, and this was not reversed in the presence of IL-2c (Supplementary Fig. 2c). Analysis of pancreas sections at the study endpoint showed clear preservation of the β-cell mass in mice treated with the combined regimen (Fig. 4d, e). A shortened treatment regimen was also effective at inhibiting diabetes development (Supplementary Fig. 3).

**Fig. 5:**
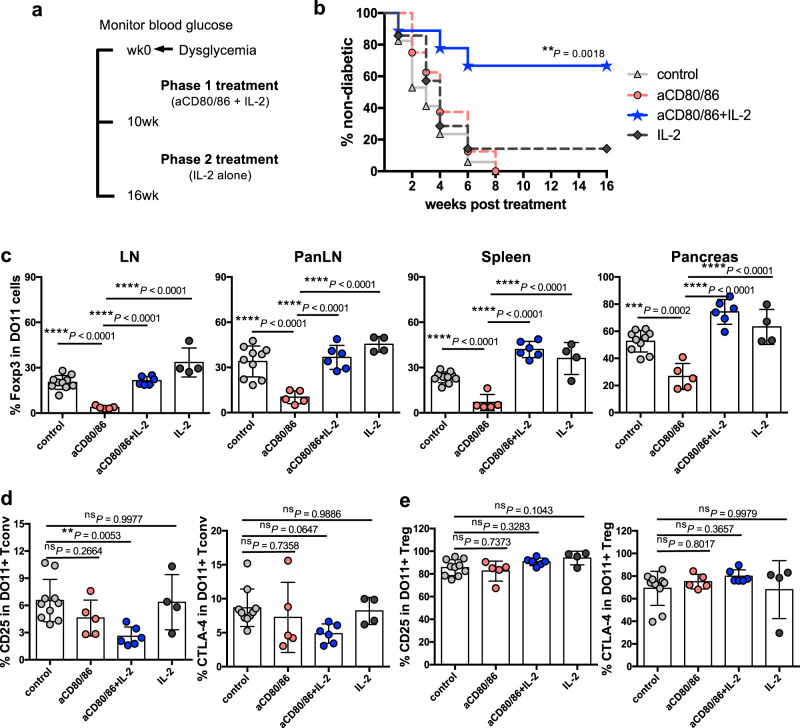
Impact of costimulation blockade and IL-2 on diabetes development in a therapeutic setting. **a** Schematic of treatment protocol. Blood glucose levels of DO11 × RIPmOVA mice were tracked, and animals with values falling between 180 mg/dL and 290 mg/dL were identified for treatment (see also Supplementary Fig. 4). Mice were injected i.p. with control Ab, anti-CD80/86 Ab, IL-2 complex or both anti-CD80/86 Ab and IL-2 complex. Anti-CD80/86 Ab was given 2 doses per week for total 10 weeks; IL-2 complex was administered in 3 doses for the first week and then 2 doses per week. **b** Percentage of non-diabetic mice based on blood glucose measurements; *n* = 17 for control, *n* = 8 for aCD80/86, *n* = 10 for aCD80/86+IL-2, *n* = 8 for IL-2. Data are collated from 3 independent experiments. Diabetes incidence in the anti-CD80/86 Ab plus IL-2 complex group, but not other treatment groups, was significantly different from the control group (*P* < 0.01, Log-rank test with Bonferroni correction). **c** Collated data showing the percentages of DO11+ Treg in peripheral lymph nodes (LN), pancreatic lymph nodes (PanLN), spleen and pancreas. **d** Collated data showing CD25 and CTLA-4 expression on gated DO11+ Tconv or **e** DO11+ Treg in the pancreatic lymph nodes. Data are presented as mean ± sd; each dot indicates one mouse. **c**–**e** *n* = 10 for control, *n* = 5 for aCD80/86, *n* = 6 for aCD80/86+IL-2, *n* = 4 for IL-2. Data are collated from 3 independent experiments. **P < 0.01, ***P < 0.001, ****P < 0.0001, ns not significant (ANOVA). Source data are provided as a Source Data file.

To test whether the combination of costimulation blockade and IL-2 was an effective treatment in a therapeutic setting, treatment was delayed until blood glucose levels had risen to 180–290 mg/dL (mean blood glucose for non-diabetic DO11+ mice is 129.9 ± 15.62 mg/dL—see Supplementary Fig. 4)(Fig. 5a). Diabetes incidence in the anti-CD80/86 Ab plus IL-2c group, but not other treatment groups, was significantly different from the control group (Fig. 5b, and Supplementary Fig. 5a). Compared with anti-CD80/86 alone, combination therapy was associated with significant increases in the proportion of Foxp3+ Treg in all tissues examined (Fig. 5c), and this skewing of the Treg:Tconv ratio was also observed when absolute cell counts of antigen-specific cells were assessed (Supplementary Fig. 5b). In the DO11 × RIPmOVA diabetes model, islet-derived OVA is known to be trafficked to the pancreatic lymph nodes (PanLN) for presentation to OVA-specific T cells^32^. In mice receiving combination therapy, antigen-specific Tconv in the PanLN showed lower expression of CD25 and a trend towards lower expression of CTLA-4, consistent with reduced activation associated with enhanced Treg suppression (Fig. 5d). Antigen-specific Treg within the same PanLN did not show a reduction in CD25 or CTLA-4 expression (Fig. 5e).

### IL-2 can preserve Treg after costimulation blockade in humanised mice

While human and mice Treg share common markers, subtle differences in their expression levels may influence the efficacy of therapeutic interventions. For example, human T cells are characterised by a continuum of CD25 expression between the Tconv and Treg compartments^33^. We therefore wished to explore how the interplay between the CD28 and IL-2 pathways influenced the homeostasis of human Treg. Specifically, we wished to test whether IL-2 could substitute for CD28 signaling in human Treg exposed to costimulation blockade. To study human immune cell subsets in an in vivo setting, we generated humanised mice by reconstitution of NOD SCID common γ-chain deficient (NSG) mice with human cord blood CD34 + cells. These animals reconstituted a human immune system with CD4 T cells that contained a Treg fraction expressing the predicted phenotypic markers (Foxp3, CD25, CTLA-4 and low CD127) (Supplementary Fig. 6).

**Fig. 6:**
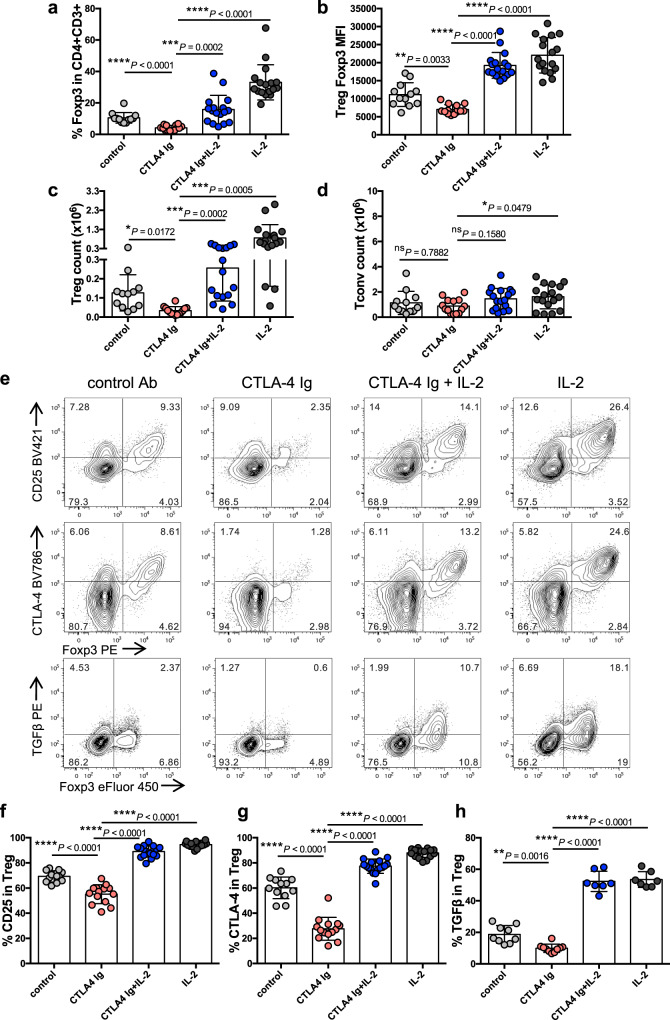
IL-2 restores Treg homeostasis after costimulation blockade in humanised mice. 4–6 week old, irradiated NSG mice (0.8 Gy) were adoptively transferred with 2 × 10^5^ CD34 + cells isolated from human cord blood. 16–23 weeks later, reconstituted humanised mice were treated with control Ab, Abatacept, IgG-(IL-2)_2_ or both Abatacept and IgG-(IL-2)_2_ as indicated. Abatacept was injected i.p. on d0, d3 and d6; IgG-(IL-2)_2_ was injected s.c. on d0, d4 and d6. IgG-(IL-2)_2_ is depicted as IL-2 in the figure for clarity. At d7, spleen cells were harvested for analysis. Graphs show collated data for the percentage Treg in gated CD4 + CD3 + cells **a**, Treg Foxp3 MFI **b**, Treg absolute number **c**, and Tconv absolute number **d**. Representative FACS plots **e**, and pooled data depict Treg expression of CD25 **f**, CTLA-4 **g**, and TGFβ **h** for each treatment group. Graphed data are presented as mean±sd; each dot indicates one mouse. (**a**–**d** and **f**, **g**) *n* = 12 for control, *n* = 14 for Abatacept, *n* = 17 for Abatacept+IL-2 and IL-2 alone; Data are collated from 5 independent experiments. **h** *n* = 9 for control, *n* = 10 for Abatacept, *n* = 7 for Abatacept+IL-2 and IL-2 alone. Data are collated from 3 independent experiments. *P < 0.05, **P < 0.01, ***P < 0.001, ****P < 0.0001, ns not significant (ANOVA). Source data are provided as a Source Data file.

To block CD28 costimulation we used Abatacept (CTLA4-Ig), which is widely used in autoimmune settings in the clinic^34^ and has been reported to impair Treg homeostasis^14–18^. IL-2 complexes are not used clinically. Instead, Roche has developed a human IL-2 molecule with a prolonged half-life as a result of fusion in bivalent stoichiometry to a non-FcγR binding human IgG1 (IgG-(IL-2)_2_)^35^. We therefore tested our combination strategy using these therapeutically relevant reagents. This experiment revealed that Abatacept significantly impaired Treg homeostasis when used alone but this was prevented if IgG-(IL-2)_2_ was also provided (Fig. 6a). In addition to restoring the percentage of Foxp3+ cells, combination therapy also abrogated the decreases in Treg Foxp3 MFI (Fig. 6b) and Treg absolute number (Fig. 6c) induced by Abatacept, while there was no significant impact on Tconv (Fig. 6d). Expression of Treg effector molecules (CD25, CTLA-4, TGFβ) was reduced by costimulation blockade but restored in the presence of IgG-(IL-2)_2_ (Fig. 6e, f, g, h). Tconv CD25 expression also appeared to increase in mice receiving IgG-(IL-2)_2_. One practical consideration related to the use of humanised mice is that each animal reconstitutes an immune system independently; thus, while we can examine splenic populations post-treatment, we cannot know whether the make-up of immune cells in the spleen was equivalent in all animals prior to treatment initiation. To offset this limitation, we examined immune cell populations in the blood, allowing both pre- and post-treatment analysis. This analysis (Supplementary Fig. 7) reinforces our conclusion that the Treg phenotypes observed in the spleen (Fig. 6) reflect the treatments rather than variable immune reconstitution. To test the combination therapy in the context of pancreatic damage, we injected humanised mice with multiple doses of low-dose streptozotocin which can trigger islet destruction in a T cell-dependent manner^36,37^. Mice subsequently treated with Abatacept and IgG-(IL-2)_2_ showed a trend towards retention of more functional beta cell mass as judged by glucose tolerance tests (Supplementary Fig. 8). Overall, these data indicate that similar principles underpin the homeostasis of human and mouse Treg providing strong rationale to test this combination strategy in the clinic.

## Discussion

The tremendous potential of Treg to suppress autoimmunity has stimulated significant interest in understanding and manipulating Treg homeostasis. An unexpected obstacle in this area has been the discovery that Treg rely on many of the same homeostatic cues as conventional effector T cells. Accordingly, inhibiting CD28 costimulation impairs the effector T cell response, but also inhibits Treg. Likewise IL-2 supplementation was originally used to stimulate effector T cells in cancer patients^38^, but turned out to be a key driver of Treg homeostasis leading to its use in autoimmune settings. This coupling of Tconv and Treg responses undoubtedly serves an important function, linking immunity and regulation and thus ensuring that immune responses are appropriately curtailed. It nevertheless presents a unique challenge for therapeutic intervention, where selective targeting of solely Tconv or solely Treg is the ultimate goal.

In this study, mechanistic understanding of how two distinct immunotherapies affect Treg has led to the identification of a combination strategy that appears to harness the beneficial aspects of both interventions. Not only does IL-2 prevent the Treg attrition seen with costimulation blockade, it also confers high expression of Treg effector molecules and strong suppressive function. Meanwhile, costimulation blockade continues to exert immune suppression in the presence of IL-2, as evidenced by the dramatic inhibition of tissue-specific autoimmunity seen in the presence of both reagents compared with IL-2 alone.

Early work in mice showing that IL-2 complexes were capable of driving Treg expansion^26,27^, and regulating autoimmunity^39,40^, paved the way for the use of this approach in the clinic^41,42^. Initially, low dose IL-2 was used to selectively target Treg, on the basis of their higher expression of IL-2Rα (CD25) and γ chains and higher inherent sensitivity to IL-2 when compared with CD4 and CD8 T cells and NK cells^43^. Recently, more refined approaches have emerged including next generation IL-2 variants^44–46^, antibodies that stabilize IL-2 in a conformation that favours Treg expansion^47^, gene-engineered T cells expressing orthogonal IL-2 and IL-2-receptor pairs^48^, CD25-biasing anti-IL-2 antibodies^49^ and IL-2/CD25 fusion proteins^50^. These approaches are designed to improve selectivity for Treg and avoid possible detrimental clinical consequences of IL-2 treatment such as activation of effector T cells and NK cells^51,52^. In some cases, they may improve sensitivity of Treg to IL-2^50^, potentially overcoming IL-2R signalling deficits associated with autoimmunity. Such therapeutics are all suitable for use in our combination strategy. Other immunomodulatory properties of the IL-2 pathway, such as its capacity to inhibit follicular helper T cells, present an added advantage in a range of autoimmune settings where these cells are implicated^53^, including type 1 diabetes^54^.

In parallel with developments in IL-2 therapeutics, new approaches to optimise costimulation blockade are also emerging, with the generation of improved CTLA-4-Ig reagents with altered affinity and prolonged half-life^55–58^, as well as reagents that target CD28 rather than its ligands^59–61^. Given that Abatacept exhibits relatively poor CD86 binding, reflecting the natural ligand affinities of native CTLA-4^62^, next-generation molecules with improved characteristics will have the benefit of suppressing Tconv activation more efficiently, but may elicit even greater Treg impairment. Furthermore, while we believe that costimulation blockade directly affects Treg homeostasis, there may be an indirect impact of reduced IL-2 secretion from Tconv, and the extent of this may differ between costimulation blockade reagents.

Notably, in both mouse and human systems, although IL-2 prevented the loss of Treg seen with costimulation blockade, Treg expansion nevertheless remained lower than with IL-2 alone. This is consistent with the work of others^63^ and suggests that although IL-2 improves the actions of Abatacept, the inverse is not the case. The ability of costimulation blockade to increase the stringency of IL-2-mediated Treg activity could conceivably be beneficial, particularly given the observed bias towards rescue of antigen-specific Treg. This suggests that such an approach might selectively support Treg populations that bear specificities relevant to the ongoing autoimmune response. As such, this provides a conceptual contrast with immunomodulation by anti-CD3 treatment, which has been suggested to broaden the repertoire of peripheral Treg by permitting specificities previously constrained by insufficient TCR engagement to undergo peripheral expansion^64^.

While costimulation blockade and IL-2 treatment constitute promising therapeutics in their own right, there are several reasons to believe that their combination might have favourable outcomes in the clinic. T cells that have lost CD28 expression would escape the effects of costimulation blockade but may be amenable to suppression by IL-2-expanded Treg. Conversely, while augmenting Treg with IL-2 alone may be sufficient for immune regulation in some settings, on other occasions the simultaneous blocking of effector T cell stimulation may be required. This may be of particular relevance in situations where costimulatory ligands are highly expressed, given that CD28 engagement has long been known to counteract Treg suppression^65^, and indeed CTLA-4-dependent Treg function operates by depriving T cells of CD28 signals^4,30^.

Combination therapies are increasingly thought to be necessary to tackle complex polygenic autoimmune diseases^66,67^. In the context of type 1 diabetes, the development of robust immune regulation strategies is particularly timely in the light of rapid advances in islet replacement technologies^68^ and stem cell derived β-cells^69^, whose potential may be limited by ongoing autoimmune responses.

## Methods

### Mice

All mice were bred and maintained in the Comparative Biology Unit at University College London or within the University of Birmingham Biological Services Unit, and all work was performed in accordance with the relevant Home Office regulations following institutional ethical approval (University of Birmingham Animal Welfare Ethical Review Body, University College London Animal Welfare Ethical Review Body). BALB/c and CD28^−/−^ mice were obtained from The Jackson Laboratory and Taconic Laboratories respectively. DO11 × RIPmOVA mice were generated by crossing DO11.10 TCR transgenic mice (purchased from The Jackson Laboratory) with RIPmOVA mice (a gift from W. Heath, the Walter and Eliza Hall Institute, Parkville, Melbourne, Australia; RIPmOVA mice express a membrane-bound form of OVA under the control of Rat Insulin Promoter). NSG mice were provided by Charles River Laboratories. Blood glucose levels were monitored using glucometer (Ascensia Elite XL, Bayer or Accu-Chek Aviva Nano, Roche). A total of 600 mg/dL is the maximum measurable reading. Mice were considered diabetic following 2 consecutive blood glucose readings of >250 mg/dL. Mice were housed in individually ventilated cages with environmental enrichment (e.g. cardboard tunnels, paper houses, chewing blocks and aspen wood wool nesting material) in a temperature- and humidity- controlled facility with a 14-h light/10-h dark cycle and ad libitum feeding. All injections were carried out in the absence of anesthesia and analgesia, typically between 10am and 4 pm, and mice were returned to the home cage immediately following the procedure. Injection of cord blood CD34 + cells was performed in the evening (typically ~9 pm). Fasting (typically 6 h) was performed in the morning and potentially edible enrichment (e.g. cardboard tunnels) was removed and replaced by non-edible enrichment (e.g. Tecniplast red plastic mouse house). The welfare of experimental animals was monitored regularly (typically immediately postprocedure, then at least every 2–3 days). No procedure-related adverse events were noted during these experiments.

### CD28 blockade in vivo experiments

6–9 week old BALB/c mice were injected i.p. with both anti-CD80 and anti-CD86 blocking Ab (clone 16–10A1 and GL-1 respectively) or control Ab (rat IgG plus hamster IgG) on d0 and d6. Abs were administered at 100 μg each per injection.

### IL-2 complex in vivo experiments

6–8 week old BALB/c and CD28^−/−^ mice were injected i.p. with IL-2 complex (0.5 μg recombinant mouse IL-2 plus 5 μg anti-mouse IL-2 Ab per injection) or control (PBS) on d0, d2, d4, d6 and d7. To make IL-2 complex, recombinant mouse IL-2 (eBioscience) and anti-mouse IL-2 Ab (clone JES6-1A12) were pre-mixed in PBS and incubated at 37 °C for 30 min. IL-2 complex was stored at 4 °C for up to 10 days following preparation.

### Short-term combination therapy using CD28 blockade and IL-2 complex

BALB/c: 7–15 week old mice were injected i.p. with control Ab, anti-CD80/86 Ab, IL-2 complex or both anti-CD80/86 Ab and IL-2 complex. Control Ab (rat IgG plus hamster IgG, 100 μg each per injection) and anti-CD80/86 Ab (clone 16–10A1 and GL-1 respectively, 100 μg each per injection) were administered on d0 and d6. IL-2 complex (0.5 μg recombinant mouse IL-2 plus 5 μg anti-mouse IL-2 Ab per injection) was given on d0, d2, d5 and d7.

DO11×RIPmOVA: 5–8 week old mice were treated i.p. with control Ab, anti-CD80/86 Ab, IL-2 complex or both anti-CD80/86 Ab and IL-2 complex. Control Ab and anti-CD80/86 Ab were administered as for BALB/c mice. 7 doses of IL-2 complex (0.5 μg recombinant mouse IL-2 plus 5 μg anti-mouse IL-2 Ab per injection) were given in total (d0, d1, d2, d3, d4, d6, and d7).

### Treatment with CD28 blockade and IL-2 complex in preventative setting

4–6 week old normoglycaemic DO11×RIPmOVA mice were treated with control Ab, anti-CD80/86 Ab, IL-2 complex or both anti-CD80/86 and IL-2 complex for 7 weeks (phase 1). Subsequently, injection of control Ab and anti-CD80/86 Ab was stopped and IL-2 complex administration continued for a further 7 weeks (phase 2). Phase 1 treatment regimen: control Ab (rat IgG plus hamster IgG, 100 μg each per injection) and anti-CD80/86 Ab (clone 16–10A1 and GL-1 respectively, 100 μg each per injection) were given at 2 doses per week. A total of 3 doses of IL-2 complex (0.5 μg recombinant mouse IL-2 plus 5 μg anti-mouse IL-2 Ab per injection) were administered in the first week followed by 2 doses per week subsequently. Phase 2 treatment regimen: IL-2 complex (0.5 μg recombinant mouse IL-2 plus 5 μg anti-mouse IL-2 Ab per injection) was administered at 2 doses per week. Blood glucose concentrations were monitored throughout the treatment period. For Fig. 4c, d, the majority of mice were culled at the 14 week timepoint; some mice in the control group were culled earlier due to diabetes induction.

### Treatment with CD28 blockade and IL-2 complex in therapeutic setting

Blood glucose levels of DO11 × RIPmOVA mice were tracked, and animals with values falling between 180 mg/dL and 290 mg/dL were identified for treatment. Mice were subsequently injected i.p. with control Ab, anti-CD80/86 Ab, IL-2 complex or both anti-CD80/86 Ab and IL-2 complex. Control Ab (rat IgG plus hamster IgG, 100 μg each per injection) and anti-CD80/86 Ab (clone 16–10A1 and GL-1 respectively, 100 μg each per injection) were given at 2 doses per week for a total of 10 weeks; IL-2 complex was administered at 3 doses for the first week and then at 2 doses per week subsequently. Blood glucose readings were monitored throughout the treatment period. Mice were culled by a Schedule 1 method 16 weeks post treatment initiation (or earlier if necessary due to diabetes induction). 2 mice were excluded in Fig. 5b because they were culled early for reasons unrelated to the experiment (indicated in Source Data).

### Generation of humanised mice and their treatment with Abatacept and IgG-(IL-2)_2_

4–6 week old NSG mice were irradiated with 0.8 Gy (X-ray) and injected i.v. with 2 × 10^5^ human CD34+ cells, isolated from cord blood using CD34 Microbead Kit UltraPure (Miltenyi Biotec). Cord blood units were obtained from Anthony Nolan Cell Therapy Centre (ANCTC). On some occasions frozen CD34 + cells were used. Tail blood samples were taken to examine human immune cell reconstitution at 16–23 weeks post cell transfer. Two mice in which circulating conventional CD4+ T cells had very low CD45RA staining (<5%) were not used for experiments. Humanised mice were treated with control Ab, Abatacept, IgG-(IL-2)_2_ or both Abatacept and IgG-(IL-2)_2_. Treatment regimen: 600 μg Abatacept in 100 μl PBS was injected i.p. on d0, d3 and d6; 4 pmole (0.7 μg) IgG-(IL-2)_2_, developed by Roche^35^, in 100 μl buffer (provided by Roche) was injected s.c. on d0, d4 and d6. At d7, spleen cells were harvested for analysis. Control Ab (human non-FcγR binding human IgG1) was also provided by Roche and injected at a 0.7 μg dose following the same injection schedule as for IgG-(IL-2)_2_.

### Flow cytometry

Mouse peripheral (inguinal, axillary and brachial) lymph node, pancreatic lymph node and spleen cells were obtained by crushing tissue through a wire mesh (Sigma-Aldrich). Red blood cells were removed from spleen and tail blood samples using lysis buffer. Lymphocytes were recovered from the pancreas by tearing the tissue into small pieces in cold PBS containing 5% FBS, 56 mM glucose (Sigma-Aldrich), 2 μg/ml Aprotinin (Roche) and 50 μg/ml TLCK (Roche). Following centrifugation, tissue was digested in pre-warmed PBS containing 304 μg/ml Liberase TL (Roche) and 10 μg/ml DNase (Sigma-Aldrich) at 37 °C for 12 min in a shaking incubator. The reaction was then quenched with cold RPMI containing 10% FBS. Following centrifugation, the sample was resuspended in warm RPMI containing 10% FBS, passed through a 40 μm cell strainer and layered onto Lympholyte-M (Tebu-bio). Lymphocytes were subsequently collected after centrifugation at 1000 g at room temperature for 20 min without brake, and washed in PBS containing 2% FBS.

Mouse cells were stained with the following Abs: CD4 PerCP (RM4-5, 1/50; BD Biosciences), Foxp3 eFluor 450 (FJK-16s, 1.5 µl; eBioscience), Ki67 FITC (B56, 1 µl; BD Biosciences), Foxp3 Pacific Blue (FJK-16s, 2 µl; eBioscience), CD4 PerCP-Cy5.5 (RM4-5, 1/50; BD Biosciences), CD3 BV785 (17A2, 1/75; BioLegend), CD25 APC (PC61, 1/100; BD Biosciences), CTLA-4 PE (UC10-4F10-11, 1 µl; BD Biosciences), CD3 BUV395 (145-2C11, 1.2 µl; BD Biosciences), TGF-β1 APC (TW7-16B4, 2.5 µl; BioLegend), CD25 PE-Cy7 (PC61.5, 1/100; eBioscience), DO11.10 TCR PE (KJ126, 1/50; eBioscience), CTLA-4 PE-CF594 (UC10-4F10-11, 1 µl; BD Biosciences) CD45 FITC (30-F11, 1/100; eBioscience), CD4 BUV737 (RM4-5, 1/100; BD Biosciences), ICOS PE-Cy7 (7E.17G9, 1/100; eBioscience), CD103 BV786 (M290, 1/200; BD Biosciences), ST2 PerCP-eFluor 710 (RMST2-2, 1/50; eBioscience), KLRG1 FITC (2F1, 1/100; eBioscience), CD39 Alexa Fluor 647 (Duha59, 1/50; BioLegend), CD73 PE (TY/11.8, 1 µl; BioLegend). Human cells were stained with the following Abs: CD45 APC (HI30, 1/100; eBioscience), CD4 Alexa Fluor 700 (RPA-T4, 3 µl; BD Biosciences), CD3 BUV395 (SK7, 3 µl; BD Biosciences), Foxp3 PE (236 A/E7, 3 µl; eBioscience), CD25 BV421 (M-A251, 3 µl; BD Biosciences), CTLA-4 BV786 (BNI3, 3ul; BD Biosciences), TGF-β1 PE (TW4-2F8, 5 µl; BioLegend), Foxp3 eFluor 450 (236 A/E7, 3ul; eBioscience), CD45RA PerCP-Cy5.5 (HI100, 1 µl; eBioscience), CD127 PE-Cy7 (ebioRDR5, 3 µl; eBioscience). Data were acquired on BD LSRFortessa (BD Biosciences) flow cytometer (majority of experiments) using BD FACSDiva v8 or v9 software or Dako CyAn ADP (Beckman Coulter) flow cytometer (4 experiments) using Summit 4.3 software and analysed using FlowJo software (v8 or v10). For gating strategy see Supplementary Fig. 9.

### Immunohistochemistry and confocal microscopy

Pancreases from treated DO11 × RIPmOVA mice were embedded in O.C.T. compound (Tissue-Tek) and frozen in liquid nitrogen vapor. Tissue sections (5–7 μm) were then cut, fixed in acetone and stored at −20 °C. For immunohistochemistry analysis, pancreas sections were stained with the following primary Abs: purified rat anti-CD4 Ab (GK1.5, 1/200 BD Biosciences), detected with sheep anti-rat biotin (1/100; The Binding Site) and followed by Vectastain ABC-AP staining kit (Vector Labs); and rabbit anti-insulin Ab (1/100; Santa Cruz), detected with goat anti-rabbit HRP (1/50; Southern Biotech). Staining was then visualized using Alkaline phosphatase and peroxidase substrates. Pancreas sections were then scanned by UCL IQPath using NDP.serve3 software. The insulin positive area and total pancreas area were quantified using QuPath software^70^. The treatment group was not revealed until after the analysis. For immunofluorescence analysis, the following primary Abs were used: purified hamster anti-CD3 (145-2C11, 1/300; BD Biosciences), guinea pig anti-insulin (1/100; Dako), rat anti-Foxp3 Alexa Fluor 488 (FJK-16s, 1/100; eBioscience); rat anti-CD45 FITC (30-F11, 1/100; eBioscience) and Rabbit anti-glucagon (1/200; Abcam). CD3 Ab was detected using goat anti-hamster Cy5 (1/60; Jackson ImmunoResearch); Insulin Ab was detected with goat anti-guinea pig Alexa Fluor 555 (1/50; Invitrogen); Rat anti-Foxp3 Alexa Fluor 488 was detected using rabbit anti-Alexa Fluor 488 (1/100; Invitrogen) followed by donkey anti-rabbit Alexa Fluor 488 (1/100; Invitrogen); rat anti-CD45 FITC Ab was detected with goat anti-FITC Alexa Fluor 488 (1/100; Invitrogen); Rabbit anti-glucagon was detected using donkey anti-rabbit Alexa Fluor 647 (1/100; Invitrogen). Pancreas sections were counterstained with DAPI (DAPI not shown in some images to increase visibility of other markers). Slides were mounted using ProLong Gold Antifade mounting medium (Invitrogen). Images were captured with a C2 + confocal microscope using NIS elements AR 4.20.02 software (Nikon) and analysed in Fiji (ImageJ).

### Statistical analysis

All statistical analysis was performed using GraphPad Prism (v6 or v8). Data were presented as mean ± sd and the *n* values are stated in the relevant Figure Legend. For two mean comparison, *P* values were calculated by two-tailed, unpaired *t* test; or two-tailed, unpaired *t* test with Welch’s correction; equality of variances was examined by F test. For the analysis of more than two means, one-way ANOVA (Dunnett’s multiple comparison test) or welch ANOVA (Dunnett’s T3 multiple comparison test) were performed; homogeneity of variances was assessed using Brown-Forsythe test. *P* values in Figs. 4c and 5b were determined by Log-rank test with Bonferroni correction. *P < 0.05, **P < 0.01, ***P < 0.001, ****P < 0.0001, *ns* not significant.

### Reporting summary

Further information on research design is available in the Nature Portfolio Reporting Summary linked to this article.

## Supplementary information

Supplementary Information

Reporting Summary

## Data Availability

The data supporting the findings of this study are available within the article and its supplementary information files. Source data are provided with this paper.

## Source data

Source Data

## References

[1] Allison JP (2015). Checkpoints. Cell.

[2] Linsley PS (1992). Immunosuppression in vivo by a soluble form of the CTLA-4 T cell activation molecule. Science.

[3] Kremer JM (2003). Treatment of rheumatoid arthritis by selective inhibition of T-cell activation with fusion protein CTLA4Ig. N. Engl. J. Med..

[4] Wing K (2008). CTLA-4 control over Foxp3+ regulatory T cell function. Science.

[5] Walker LS (2013). Treg and CTLA-4: Two intertwining pathways to immune tolerance. J. Autoimmun..

[6] Kurup SP (2017). Regulatory T cells impede acute and long-term immunity to blood-stage malaria through CTLA-4. Nat. Med..

[7] Kuehn HS (2014). Immune dysregulation in human subjects with heterozygous germline mutations in CTLA4. Science.

[8] Schubert D (2014). Autosomal dominant immune dysregulation syndrome in humans with CTLA4 mutations. Nat. Med..

[9] Simpson TR (2013). Fc-dependent depletion of tumor-infiltrating regulatory T cells co-defines the efficacy of anti-CTLA-4 therapy against melanoma. J. Exp. Med..

[10] Salomon B (2000). B7/CD28 costimulation is essential for the homeostasis of the CD4+CD25+ immunoregulatory T cells that control autoimmune diabetes. Immunity.

[11] Riella LV (2012). Deleterious effect of CTLA4-Ig on a Treg-dependent transplant model. Am. J. Transpl..

[12] Yang J (2009). Paradoxical functions of B7: CD28 costimulation in a MHC class II-mismatched cardiac transplant model. Am. J. Transpl..

[13] Tang Q (2003). Cutting edge: CD28 controls peripheral homeostasis of CD4+CD25+ regulatory T cells. J. Immunol..

[14] Pieper J (2013). CTLA4-Ig (abatacept) therapy modulates T cell effector functions in autoantibody-positive rheumatoid arthritis patients. BMC Immunol..

[15] Alvarez-Quiroga C (2011). CTLA-4-Ig therapy diminishes the frequency but enhances the function of Treg cells in patients with rheumatoid arthritis. J. Clin. Immunol..

[16] Orban T (2014). Reduction in CD4 central memory T-cell subset in costimulation modulator abatacept-treated patients with recent-onset type 1 diabetes is associated with slower C-peptide decline. Diabetes.

[17] Salomon S (2017). Th17 and CD24(hi)CD27(+) regulatory B lymphocytes are biomarkers of response to biologics in rheumatoid arthritis. Arthritis Res Ther..

[18] Szentpetery A (2017). Abatacept reduces synovial regulatory T-cell expression in patients with psoriatic arthritis. Arthritis Res Ther..

[19] Sansom DM, Walker LS (2006). The role of CD28 and cytotoxic T-lymphocyte antigen-4 (CTLA-4) in regulatory T-cell biology. Immunol. Rev..

[20] Setoguchi R, Hori S, Takahashi T, Sakaguchi S (2005). Homeostatic maintenance of natural Foxp3(+) CD25(+) CD4(+) regulatory T cells by interleukin (IL)−2 and induction of autoimmune disease by IL-2 neutralization. J. Exp. Med..

[21] Fontenot JD, Rasmussen JP, Gavin MA, Rudensky AY (2005). A function for interleukin 2 in Foxp3-expressing regulatory T cells. Nat. Immunol..

[22] D’Cruz LM, Klein L (2005). Development and function of agonist-induced CD25+Foxp3+ regulatory T cells in the absence of interleukin 2 signaling. Nat. Immunol..

[23] Edner NM (2020). Follicular helper T cell profiles predict response to costimulation blockade in type 1 diabetes. Nat. Immunol..

[24] Yamanouchi J (2007). Interleukin-2 gene variation impairs regulatory T cell function and causes autoimmunity. Nat. Genet..

[25] Chinen T (2016). An essential role for the IL-2 receptor in Treg cell function. Nat. Immunol..

[26] Boyman O, Kovar M, Rubinstein MP, Surh CD, Sprent J (2006). Selective stimulation of T cell subsets with antibody-cytokine immune complexes. Science.

[27] Webster KE (2009). In vivo expansion of T reg cells with IL-2-mAb complexes: induction of resistance to EAE and long-term acceptance of islet allografts without immunosuppression. J. Exp. Med..

[28] Spangler JB (2015). Antibodies to interleukin-2 elicit selective T cell subset potentiation through distinct conformational mechanisms. Immunity.

[29] Wesley JD, Sather BD, Perdue NR, Ziegler SF, Campbell DJ (2010). Cellular requirements for diabetes induction in DO11.10xRIPmOVA mice. J. Immunol..

[30] Qureshi OS (2011). Trans-endocytosis of CD80 and CD86: a molecular basis for the cell-extrinsic function of CTLA-4. Science.

[31] Orban T (2011). Co-stimulation modulation with abatacept in patients with recent-onset type 1 diabetes: a randomised, double-blind, placebo-controlled trial. Lancet.

[32] Walker LS, Chodos A, Eggena M, Dooms H, Abbas AK (2003). Antigen-dependent proliferation of CD4+ CD25+ regulatory T cells in vivo. J. Exp. Med.

[33] Miyara M (2009). Functional delineation and differentiation dynamics of human CD4+ T cells expressing the FoxP3 transcription factor. Immunity.

[34] Edner NM, Carlesso G, Rush JS, Walker LSK (2020). Targeting co-stimulatory molecules in autoimmune disease. Nat. Rev. Drug Disco..

[35] Bell CJ (2015). Sustained in vivo signaling by long-lived IL-2 induces prolonged increases of regulatory T cells. J. Autoimmun..

[36] Kantwerk G, Cobbold S, Waldmann H, Kolb H (1987). L3T4 and Lyt-2 T cells are both involved in the generation of low-dose streptozotocin-induced diabetes in mice. Clin. Exp. Immunol..

[37] Herold KC (1992). Prevention of autoimmune diabetes with nonactivating anti-CD3 monoclonal antibody. Diabetes.

[38] Rosenberg SA (2014). IL-2: the first effective immunotherapy for human cancer. J. Immunol..

[39] Tang Q (2008). Central role of defective interleukin-2 production in the triggering of islet autoimmune destruction. Immunity.

[40] Grinberg-Bleyer Y (2010). IL-2 reverses established type 1 diabetes in NOD mice by a local effect on pancreatic regulatory T cells. J. Exp. Med..

[41] Saadoun D (2011). Regulatory T-cell responses to low-dose interleukin-2 in HCV-induced vasculitis. N. Engl. J. Med..

[42] Koreth J (2011). Interleukin-2 and regulatory T cells in graft-versus-host disease. N. Engl. J. Med..

[43] Yu A (2015). Selective IL-2 responsiveness of regulatory T cells through multiple intrinsic mechanisms supports the use of low-dose IL-2 therapy in type 1 diabetes. Diabetes.

[44] Mitra S (2015). Interleukin-2 activity can be fine tuned with engineered receptor signaling clamps. Immunity.

[45] Peterson, L. B. et al. A long-lived IL-2 mutein that selectively activates and expands regulatory T cells as a therapy for autoimmune disease. *J. Autoimmun.* (2018). 10.1016/j.jaut.2018.10.017 PMC6284106 30446251

[46] Khoryati, L. et al. An IL-2 mutein engineered to promote expansion of regulatory T cells arrests ongoing autoimmunity in mice. *Sci. Immunol.* **5** (2020). 10.1126/sciimmunol.aba5264 PMC7643170 32817295

[47] Trotta E (2018). A human anti-IL-2 antibody that potentiates regulatory T cells by a structure-based mechanism. Nat. Med..

[48] Sockolosky JT (2018). Selective targeting of engineered T cells using orthogonal IL-2 cytokine-receptor complexes. Science.

[49] Karakus, U. et al. Receptor-gated IL-2 delivery by an anti-human IL-2 antibody activates regulatory T cells in three different species. *Sci. Transl. Med.* **12** (2020). 10.1126/scitranslmed.abb9283 33328333

[50] Ward NC (2020). Persistent IL-2 receptor signaling by IL-2/CD25 fusion protein controls diabetes in NOD mice by multiple mechanisms. Diabetes.

[51] Long SA (2012). Rapamycin/IL-2 combination therapy in patients with type 1 diabetes augments Tregs yet transiently impairs beta-cell function. Diabetes.

[52] Long SA, Buckner JH, Greenbaum CJ (2013). IL-2 therapy in type 1 diabetes: “Trials” and tribulations. Clin. Immunol..

[53] Ueno H (2016). T follicular helper cells in human autoimmunity. Curr. Opin. Immunol..

[54] Kenefeck R (2015). Follicular helper T cell signature in type 1 diabetes. J. Clin. Investig..

[55] Song L (2017). ASP2409, a next-generation CTLA4-Ig, versus belatacept in renal allograft survival in cynomolgus monkeys. Am. J. Transpl..

[56] Douthwaite J (2017). A CD80-biased CTLA4-Ig fusion protein with superior in vivo efficacy by simultaneous engineering of affinity, selectivity, stability, and FcRn binding. J. Immunol..

[57] Bernett MJ (2013). Immune suppression in cynomolgus monkeys by XPro9523: an improved CTLA4-Ig fusion with enhanced binding to CD80, CD86 and neonatal Fc receptor FcRn. mAbs.

[58] Larsen CP (2005). Rational development of LEA29Y (belatacept), a high-affinity variant of CTLA4-Ig with potent immunosuppressive properties. Am. J. Transpl..

[59] Watkins BK (2018). CD28 blockade controls T cell activation to prevent graft-versus-host disease in primates. J. Clin. Invest..

[60] Poirier N (2016). First-in-human study in healthy subjects with FR104, a pegylated monoclonal antibody fragment antagonist of CD28. J. Immunol..

[61] Badell IR (2018). Selective CD28 blockade results in superior inhibition of donor-specific T follicular helper cell and antibody responses relative to CTLA4-Ig. Am. J. Transpl..

[62] Collins AV (2002). The interaction properties of costimulatory molecules revisited. Immunity.

[63] Charbonnier LM (2012). CTLA4-Ig restores rejection of MHC class-II mismatched allografts by disabling IL-2-expanded regulatory T cells. Am. J. Transpl..

[64] Nishio J, Feuerer M, Wong J, Mathis D, Benoist C (2010). Anti-CD3 therapy permits regulatory T cells to surmount T cell receptor-specified peripheral niche constraints. J. Exp. Med..

[65] Takahashi T (1998). Immunologic self-tolerance maintained by CD25+CD4+ naturally anergic and suppressive T cells: induction of autoimmune disease by breaking their anergic/suppressive state. Int. Immunol..

[66] Fugger L, Jensen LT, Rossjohn J (2020). Challenges, progress, and prospects of developing therapies to treat autoimmune diseases. Cell.

[67] Long SA, Speake C (2021). Combination therapy in recent-onset type 1 diabetes. Lancet Diabetes Endocrinol..

[68] Yoshihara E (2020). Immune-evasive human islet-like organoids ameliorate diabetes. Nature.

[69] Hogrebe NJ, Maxwell KG, Augsornworawat P, Millman JR (2021). Generation of insulin-producing pancreatic beta cells from multiple human stem cell lines. Nat. Protoc..

[70] Bankhead P (2017). QuPath: open source software for digital pathology image analysis. Sci. Rep..

