# Author Correction: Costimulation blockade in combination with IL-2 permits regulatory T cell sparing immunomodulation that inhibits autoimmunity

**DOI:** 10.1038/s41467-023-41483-4

**Published:** 2023-09-15

**Authors:** Chun Jing Wang, Lina Petersone, Natalie M. Edner, Frank Heuts, Vitalijs Ovcinnikovs, Elisavet Ntavli, Alexandros Kogimtzis, Astrid Fabri, Yassin Elfaki, Luke P. Houghton, Ralf J. Hosse, David A. Schubert, Andreas P. Frei, Ellen M. Ross, Lucy S. K. Walker

**Affiliations:** 1 Institute of Immunity & Transplantation, Pears Building, University College London Division of Infection & Immunity, London, UK; 2 Roche Innovation Center Zurich, Roche Pharma Research & Early Development (pRED), Schlieren, Switzerland; 3 Roche Innovation Center Basel, Roche Pharma Research & Early Development (pRED), Basel, Switzerland

**Keywords:** Peripheral tolerance, Immunosuppression, Regulatory T cells, Interleukins

Correction to: *Nature Communications* 10.1038/s41467-022-34477-1, published online 09 November 2022

The original version of this article contained an error in the Methods section which incorrectly omitted “Cord blood units were obtained from Anthony Nolan Cell Therapy Centre (ANCTC).” From page 9, Method section, subsection “Generation of humanised mice and their treatment with Abatacept and IgG-(IL-2)_2_”.

This now reads “….CD34 Microbead Kit UltraPure (Miltenyi Biotec). Cord blood units were obtained from Anthony Nolan Cell Therapy Centre (ANCTC). On some occasions…”

This has been corrected in both the PDF and HTML versions of the article.

