# Supplementary material for: Costimulation blockade in combination with IL-2 permits regulatory T cell sparing immunomodulation that inhibits autoimmunity
Source: Nat Commun. 2022 Nov 9;13:6757. doi: 10.1038/s41467-022-34477-1 (PMC9643453; doi:10.1038/s41467-022-34477-1)
Supplement: Supplementary file 1 — Supplementary Information [file 41467_2022_34477_MOESM1_ESM.pdf]

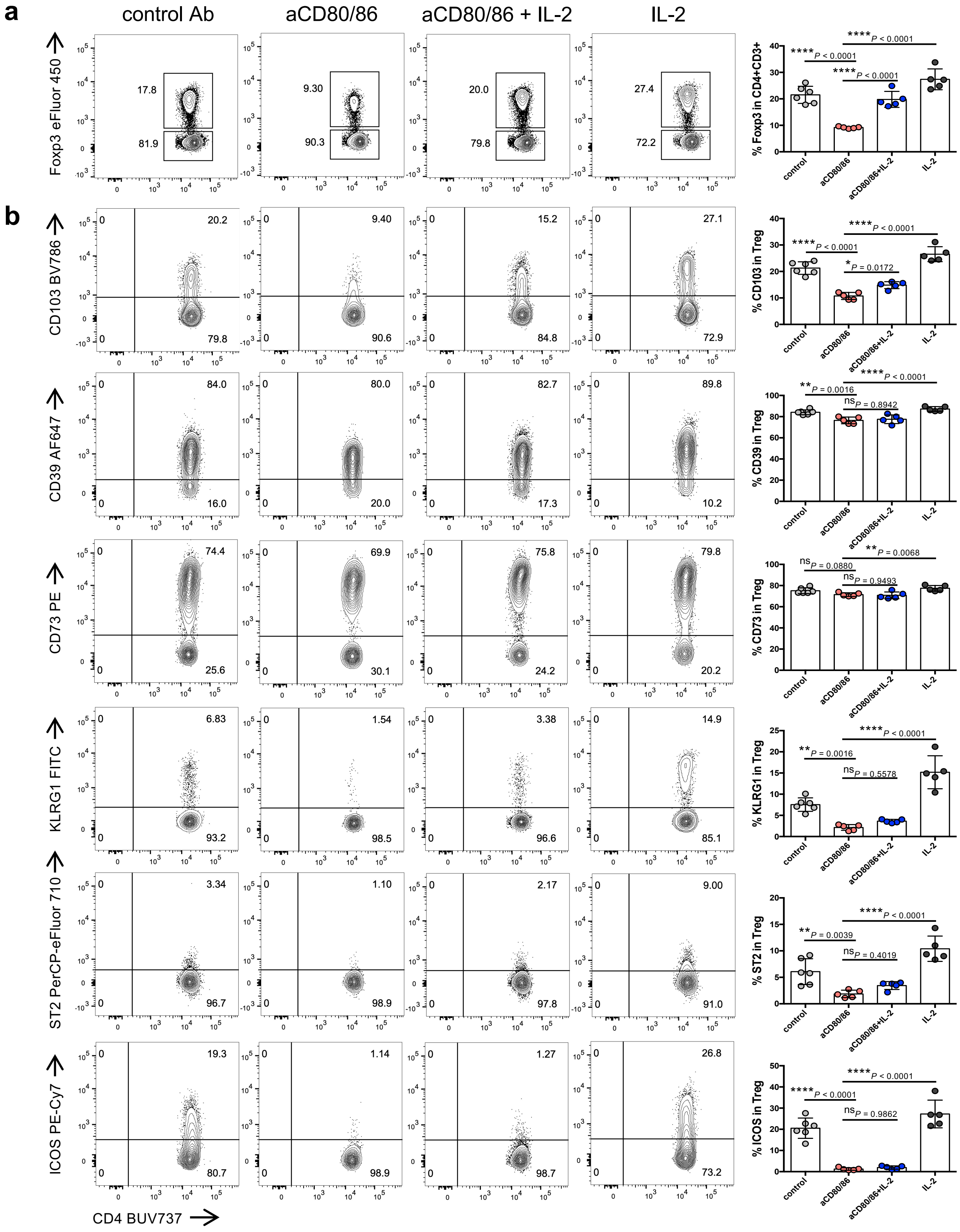

Supplementary Figure 1 Additional phenotypic data on Treg from mice treated with costimulation blockade and/or IL-2 complex. 8-10 week old BALB/c mice were injected i.p. with control Ab, anti-CD80/86 Ab, IL-2 complex or both as indicated (anti-CD80/86 Ab was injected on d0 and d6; IL-2 complex was injected on d0, d2, d5 and d7). Spleen cells were analysed by flow cytometry for expression of the indicated markers at d8. (**a**) Representative plots and collated data showing Foxp3 expression in gated CD3+CD4+ cells. (**b**) Representative plots and collated data showing expression of CD103, CD39, CD73, KLRG1, ST2 or ICOS in gated CD3+CD4+Foxp3+ cells. Graphs show mean±s.d.; each dot indicates one mouse (n=5-6 per group). Data are collated from 3 independent experiments. * P < 0.05, ** P < 0.01, **** P < 0.0001, ns=not significant (ANOVA). Treg were also assessed for IL-10 expression (with or without anti-CD3/CD28 bead stimulation) however expression was low (<2.5% Treg) and did not differ between conditions so is not depicted. Source data are provided as a Source Data file.

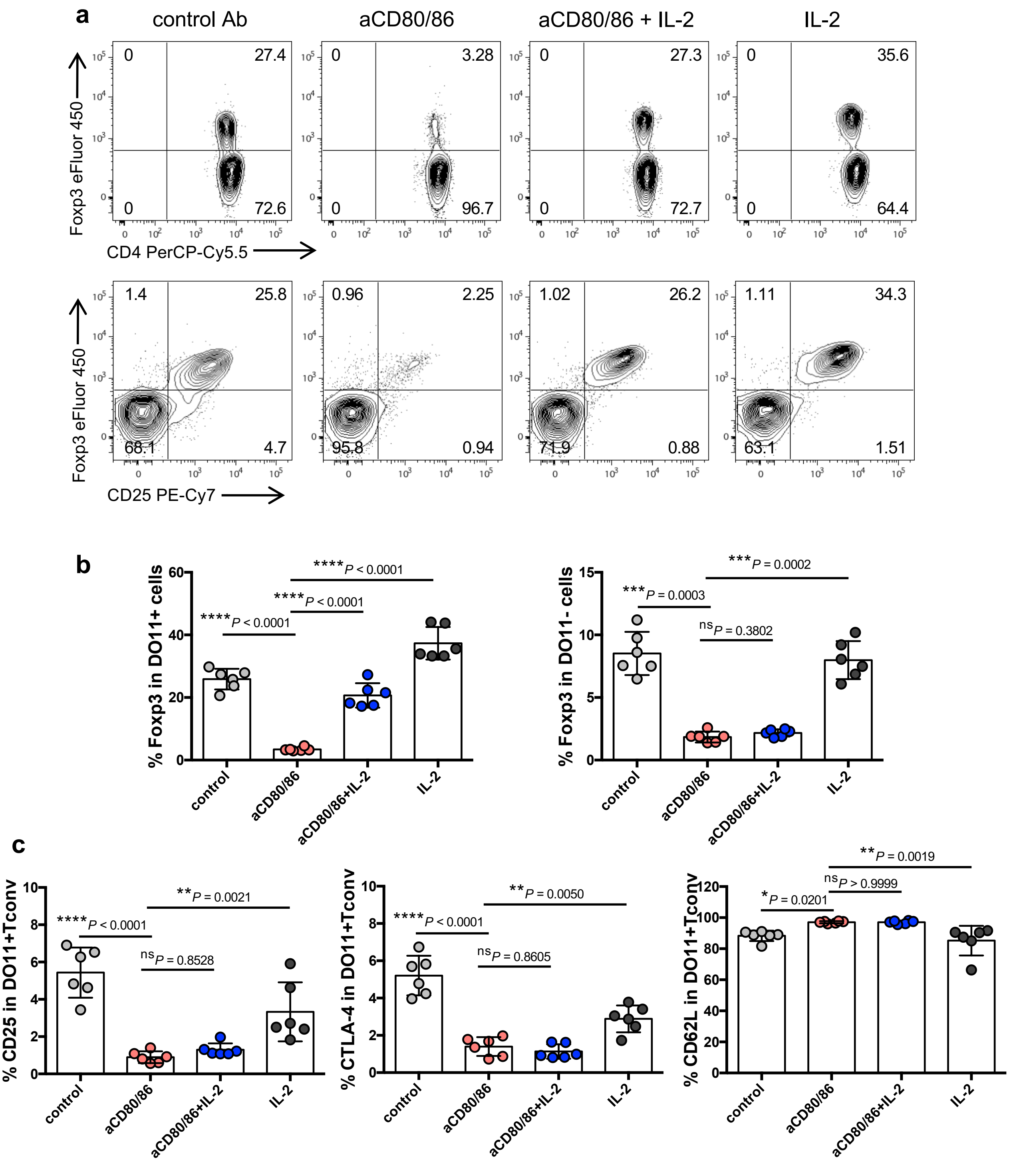

Supplementary Figure 2. Co-administration of IL-2 selectively counteracts the detrimental effect of CD28 blockade on islet-specific Treg. 4-6 week old normoglycaemic DO11 x RIPmOVA mice were treated with control Ab, anti-CD80/86 Ab, IL-2 complex or both anti-CD80/86 Ab and IL-2 complex using the treatment protocol described in **Figure 4**. 6-7 weeks post treatment initiation, tail blood samples were taken for FACS analysis. (**a**) Representative plots showing staining for CD4, intracellular Foxp3 and CD25 in gated DO11+ cells (CD4+CD3+DO11+). (**b**) Collated data showing the percentage of Treg in gated DO11+ cells (CD4+CD3+DO11+) (left) or DO11- cells (CD4+CD3+DO11-) (right). (**c**) Collated data showing the percentage of CD25, CTLA-4 or CD62L in gated DO11+ conventional T cells (CD4+CD3+DO11+Foxp3-). Data are presented as mean±s.d. (n=6 for each group); each dot indicates one mouse. (**a-c**) Data are collated from 3 independent experiments. * $P < 0.05$, ** $P < 0.01$, *** $P < 0.001$, **** $P < 0.0001$, ns=not significant (ANOVA). Source data are provided as a Source Data file.

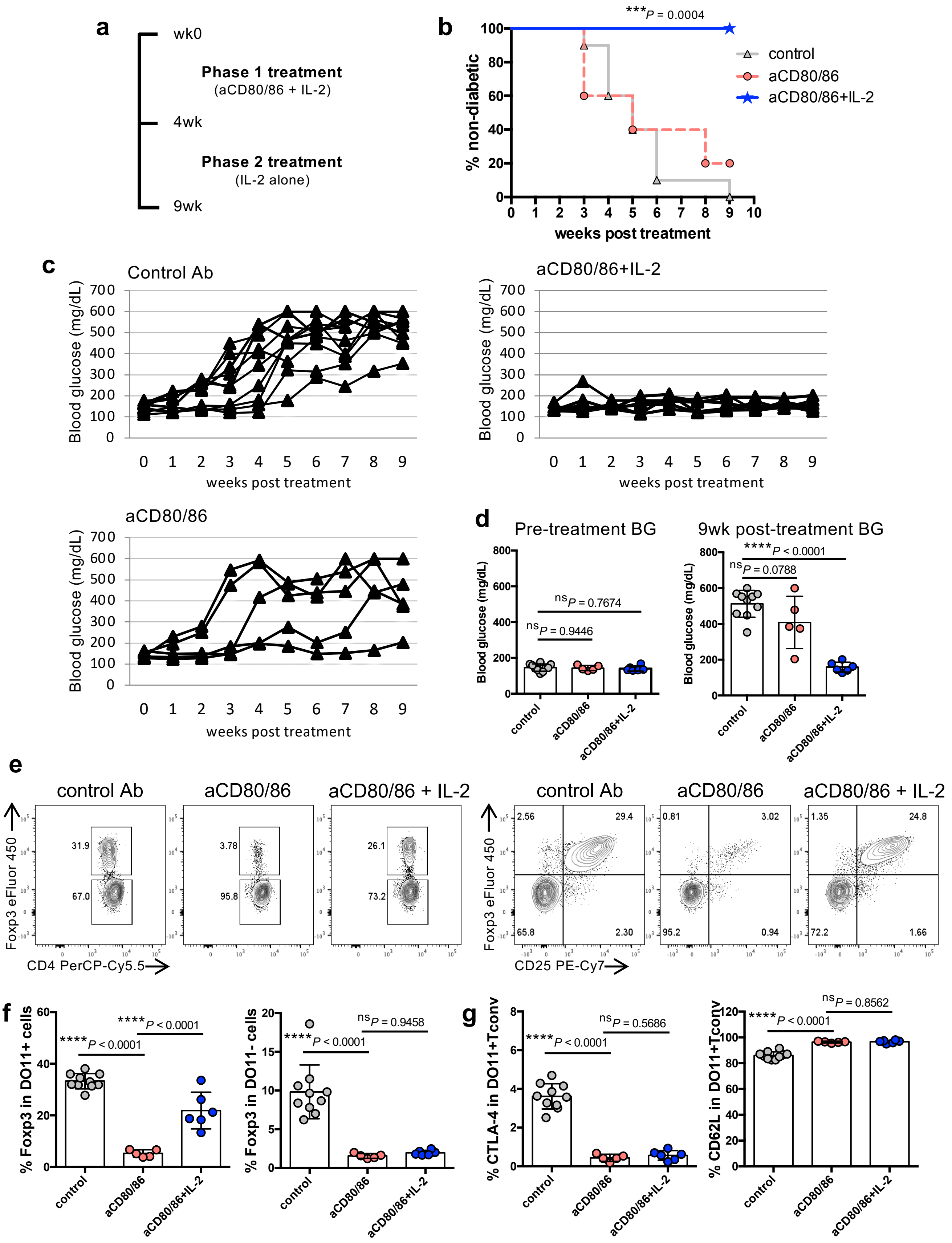

a
wk0
Phase 1 treatment
(aCD80/86 + IL-2)
4wk
Phase 2 treatment
(IL-2 alone)
9wk
b
***P = 0.0004
% non-diabetic
weeks post treatment
control
aCD80/86
aCD80/86+IL-2
c
Control Ab
aCD80/86+IL-2
aCD80/86
Blood glucose (mg/dL)
weeks post treatment
d
Pre-treatment BG
9wk post-treatment BG
nsP = 0.7674
nsP = 0.9446
****P < 0.0001
nsP = 0.0788
Blood glucose (mg/dL)
control
aCD80/86
aCD80/86+IL-2
e
control Ab
aCD80/86
aCD80/86 + IL-2
Foxp3 eFluor 450
CD4 PerCP-Cy5.5
CD25 PE-Cy7
31.9
67.0
3.78
95.8
26.1
73.2
2.56
29.4
65.8
2.30
0.81
3.02
95.2
0.94
1.35
24.8
72.2
1.66
f
% Foxp3 in DO11+ cells
****P < 0.0001
****P < 0.0001
% Foxp3 in DO11- cells
****P < 0.0001
nsP = 0.9458
g
% CTLA-4 in DO11+Tconv
****P < 0.0001
nsP = 0.5686
% CD62L in DO11+Tconv
****P < 0.0001
nsP = 0.8562
control
aCD80/86
aCD80/86+IL-2

Supplementary Figure 3 Impact of costimulation blockade and IL-2 on diabetes development using a shortened treatment protocol. (**a**) 4-5 week old normoglycaemic DO11 x RIPmOVA mice were treated with anti-CD80/86 Ab plus IL-2 complex for 4 weeks (phase 1). Subsequently, mice were maintained on IL-2 complex alone (phase 2). Control groups received either anti-CD80/86 alone or control Ab (not depicted). (**b**) Percentage of non-diabetic mice based on blood glucose measurements (n=10 for control, n=5 for anti-CD80/86, n=6 for anti-CD80/86+IL-2). Diabetes incidence in the anti-CD80/86 Ab plus IL-2 complex group, but not the anti-CD80/86 Ab group was significantly different from the control group (****P*=0.0004, Log-rank test with Bonferroni correction). (**c**) Blood glucose readings for individual mice in each treatment group. (**d**) Collated data showing blood glucose readings for mice allocated to each experimental group before (left) or 9wk after (right) treatment. (**e-g**) Analysis of tail blood samples 4 weeks post treatment initiation. (**e**) Representative plots show staining for CD4 and intracellular Foxp3 (left), or CD25 and intracellular Foxp3 (right) in gated DO11+ cells (CD4+CD3+DO11+). (**f**) Collated data showing the percentage of Treg in gated DO11+ cells (CD4+CD3+DO11+) (left) or DO11- cells (CD4+CD3+DO11-) (right). (**g**) Collated data showing the percentage of DO11+ conventional T cells (CD4+CD3+DO11+Foxp3-) expressing CTLA-4 or CD62L. (**d, f-g**) Data are presented as mean±s.d.; each dot indicates one mouse. n=10 for control, n=5 for anti-CD80/86, n=6 for anti-CD80/86+IL-2. (**b-g**) Data are collated from 2 independent experiments. *** $P < 0.001$, **** $P < 0.0001$, ns=not significant (ANOVA). Source data are provided as a Source Data file.

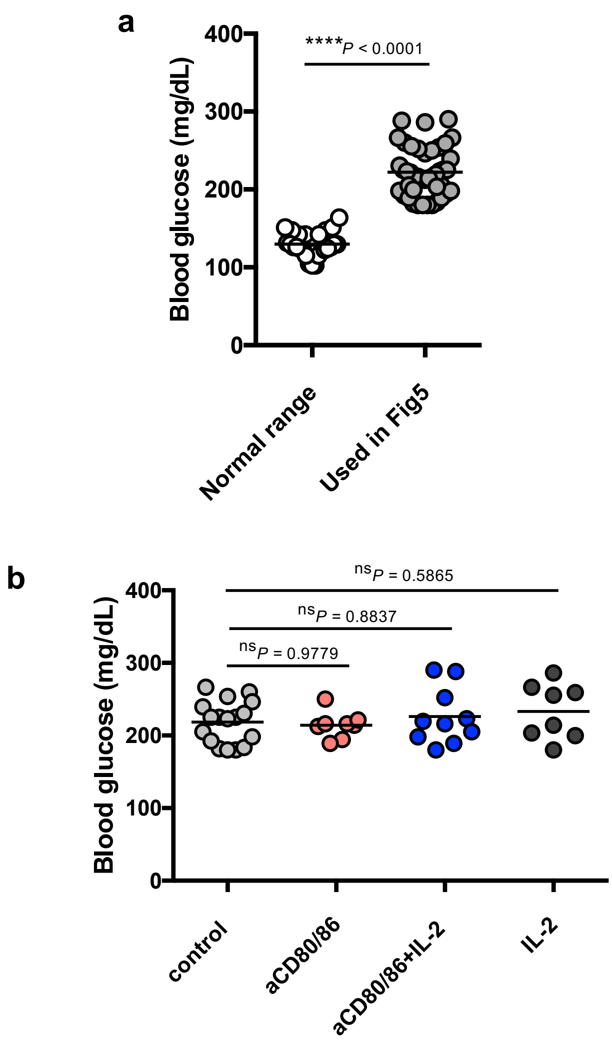

Supplementary Figure 4. Comparison of normal blood glucose readings from non-diabetic mice with readings from mice tested in the therapeutic setting in **Figure 5**. (**a**) Collated data showing blood glucose readings of non-diabetic DO11+ mice (n=26) and the initial blood glucose readings of the DO11 x RIPmOVA mice used in **Figure 5** (n=43). Mean values are shown; each dot indicates one mouse. **** $P < 0.0001$ (two-tailed *t* test). (**b**) The initial blood glucose readings of DO11 x RIPmOVA mice allocated to each experimental group showing that there were no significant differences prior to treatment initiation. Mean values are shown; each dot indicates one mouse. n= 17 for control, n=8 for aCD80/86, n=10 for aCD80/86+IL-2, n=8 for IL-2. Data are collated from 3 independent experiments. ns=not significant (ANOVA). Source data are provided as a Source Data file.

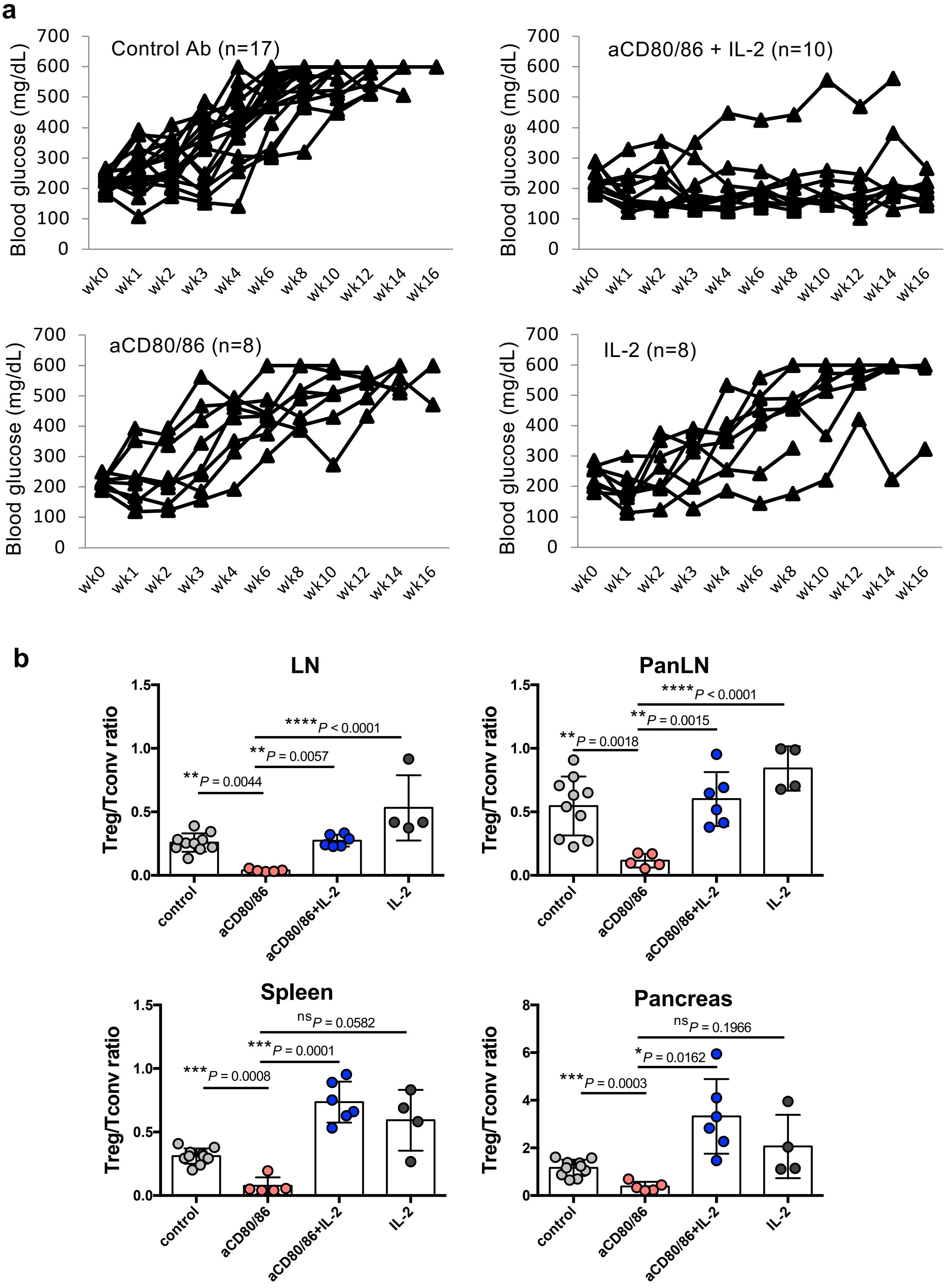

Supplementary Figure 5. Collated diabetes data and ratio of antigen-specific Treg/Tconv for mice tested in the therapeutic setting in **Figure 5**. Blood glucose levels of DO11 x RIPmOVA mice were tracked, and animals with values falling between 180 mg/dL and 290 mg/dL were identified and treated with control Ab, anti-CD80/86 Ab, IL-2 complex or both anti-CD80/86 Ab and IL-2 complex as detailed in the methods. (**a**) Blood glucose readings of DO11 x RIPmOVA mice treated with control Ab (top left), anti-CD80/86 Ab plus IL-2 (top right), anti-CD80/86 Ab alone (bottom left) or IL-2 alone (bottom right). n=17 for control, n=8 for aCD80/86, n=10 for aCD80/86+IL-2, n=8 for IL-2. Data are collated from 3 independent experiments. (**b**) Collated data show the ratios of DO11+ Treg to DO11+ Tconv based on absolute cell counts in peripheral lymph nodes (LN), pancreatic lymph nodes (PanLN), spleen and pancreas. Data are presented as mean±s.d.; each dot indicates one mouse. n=10 for control, n=5 for aCD80/86, n=6 for aCD80/86+IL-2, n=4 for IL-2. Data are collated from 3 independent experiments. * $P < 0.05$, ** $P < 0.01$, *** $P < 0.001$, **** $P < 0.0001$, ns=not significant (ANOVA). Source data are provided as a Source Data file.

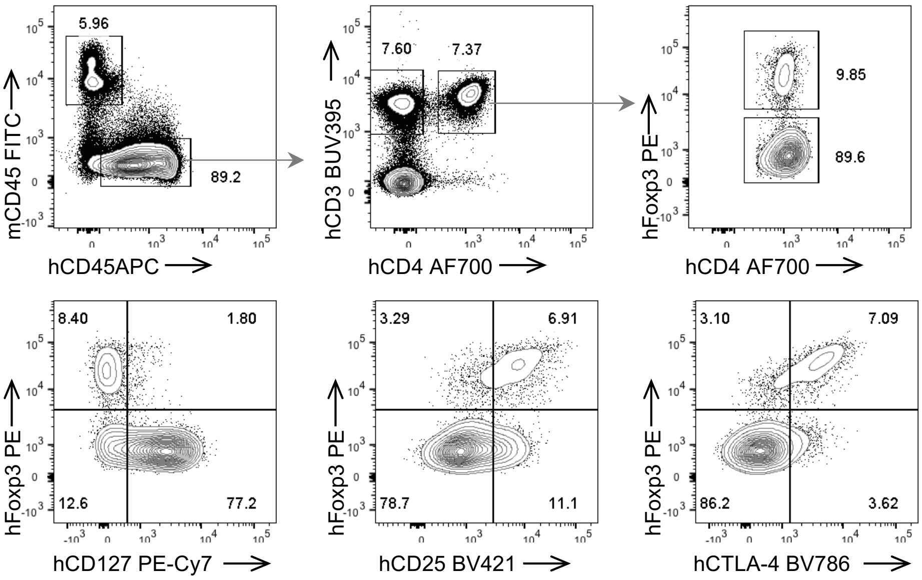

Supplementary Figure 6. Reconstitution of human Tregs in NSG mice injected with human cord blood CD34+ cells. 4-6 week old irradiated (0.8Gy) NSG mice were adoptively transferred with $2x10^5$ CD34+ cells isolated from human cord blood. 16-23 weeks later, spleens cells were harvested for analysis. FACS plots show representative staining for human CD45 (hCD45), mouse CD45 (mCD45), human CD3 (hCD3), human CD4 (hCD4), intracellular human Foxp3 (hFoxp3), intracellular human CTLA-4 (hCTLA-4), human CD25 (hCD25) and human CD127 (hCD127). Lower plots were gated on CD4+CD3+ cells. The human Foxp3+ Tregs exhibit the expected $CD25^+CTLA\text{-}4^{hi}CD127^{low}$ phenotype. Data are representative of 3 independent experiments.

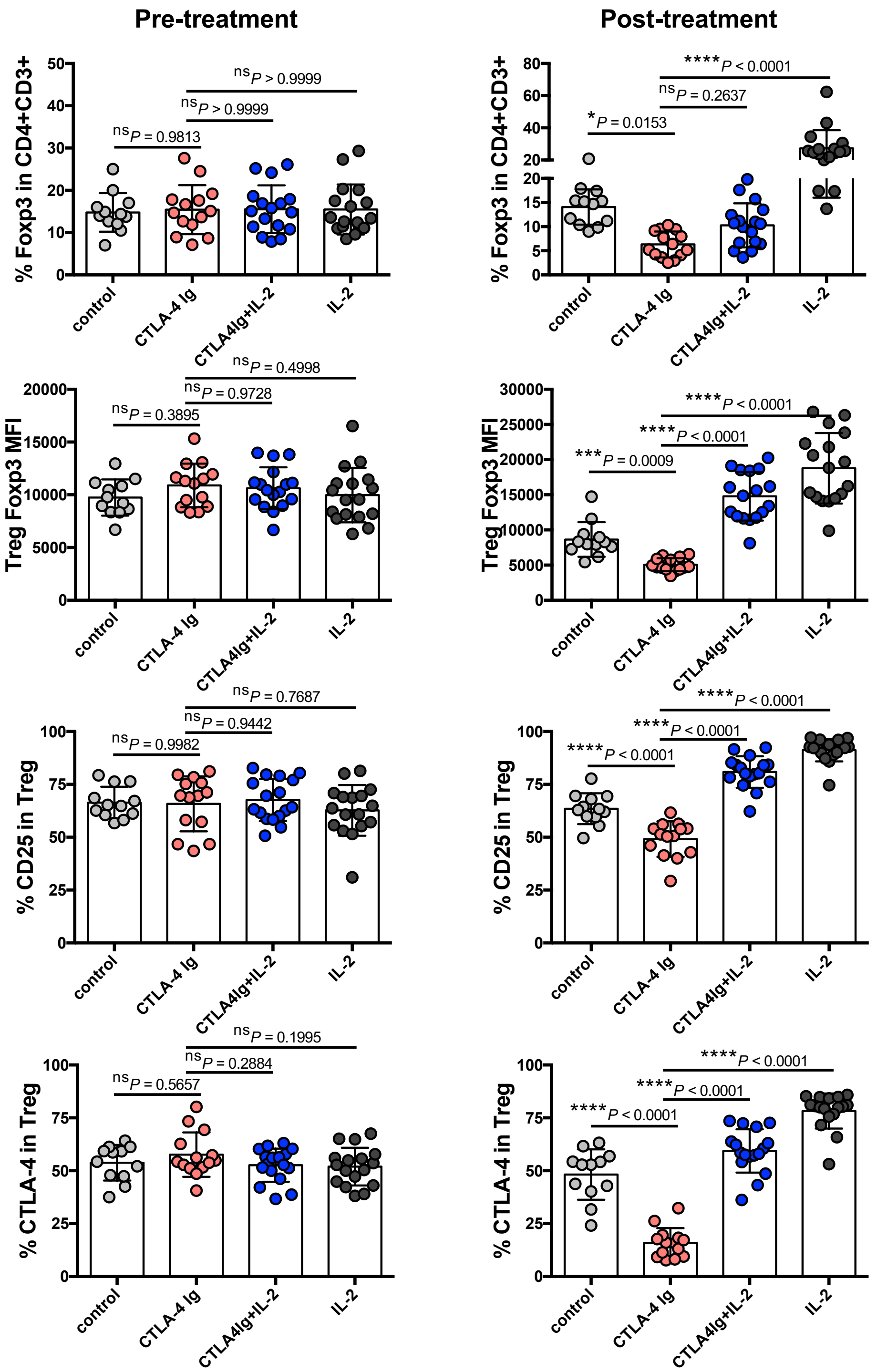

Supplementary Figure 7. Analysis of pre and post-treatment blood samples in humanised mice. 4-6 week old irradiated (0.8Gy) NSG mice were adoptively transferred with $2x10^5$ CD34+ cells isolated from human cord blood. 16-23 weeks later, reconstituted humanised mice were treated with control Ab, Abatacept, IgG-(IL-2)$_2$ or both Abatacept and IgG-(IL-2)$_2$ as indicated. Abatacept was injected i.p. on d0, d3 and d6; IgG-(IL-2)$_2$ was injected s.c. on d0, d4 and d6. IgG-(IL-2)$_2$ is depicted as IL-2 in the figure for clarity. Blood samples were taken for FACS analysis prior to treatment (Pre-treatment) and 7 days after treatment initiation (Post-treatment). Graphs show collated data for the percentage of Foxp3+ cells in gated CD4+CD3+ cells, the MFI of Foxp3 within the CD4+Foxp3+ Treg gate, the percentage of CD4+Foxp3+ cells expressing CD25 and the percentage of CD4+Foxp3+ cells expressing CTLA-4. Data are presented as mean±s.d.; each dot indicates one mouse. n=12 for control, n=14 for aCD80/86, n=17 for aCD80/86+IL-2, n=17 for IL-2. Data are collated from 5 independent experiments. * P < 0.05, *** P < 0.001, **** P < 0.0001, ns=not significant (ANOVA). Source data are provided as a Source Data file.

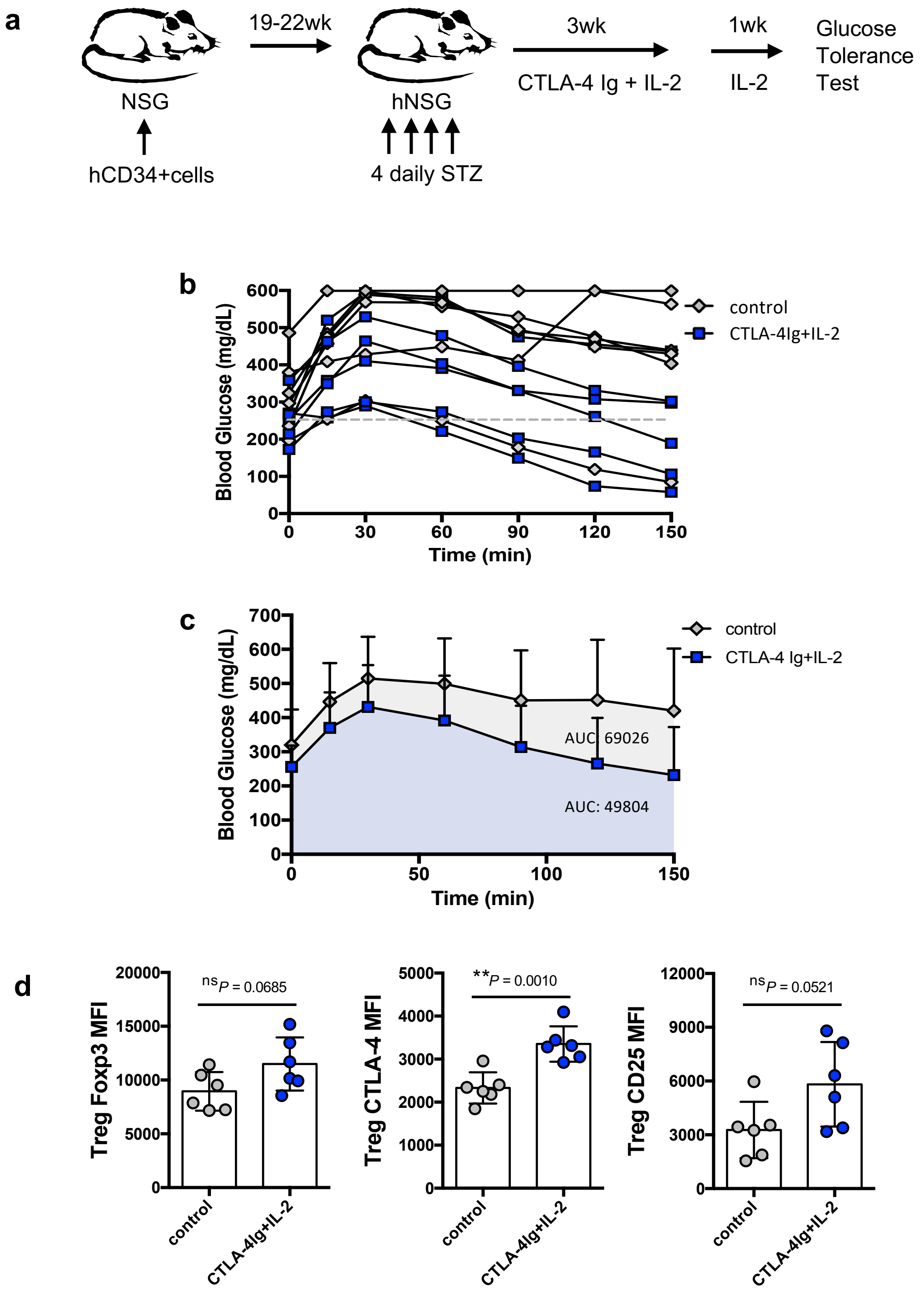

Supplementary Figure 8 Impact of co-stimulation blockade and IL-2 on low dose STZ-induced diabetes in humanised mice. (**a**) Timeline of experimental protocol. 4-6 week old, irradiated (0.8Gy) NSG mice were adoptively transferred with 2x10^5 CD34+ human cord blood cells and left to reconstitute for 19-22 weeks. Reconstituted mice (hNSG) were injected i.p. with 40mg/kg STZ in citrate buffer daily for 4 days to induce diabetes (mice were fasted prior to the first dose of STZ). 1 mouse did not develop diabetes (blood glucose <200mg/dL by d9) and was excluded from further analysis. From d5, mice were treated with control Ab or Abatacept (CTLA-4-Ig) and IgG-(IL-2)$_2$ (IL-2). Abatacept was injected i.p. twice per week for 3 weeks; IgG-(IL-2)$_2$ was injected s.c. twice per week for 4 weeks. (**b-d**) A glucose tolerance test (GTT) was then performed (injection of fasted mice with 1g/kg glucose i.p.) and spleen cells were harvested for flow cytometry analysis. Data are collated from 2 independent experiments. Graphs show blood glucose levels during GTT for each individual mouse (**b**) or collated for each group and presented as means+s.d. and AUC (**c**). (**d**) Collated flow cytometry data showing MFI of Foxp3, CTLA-4 or CD25 in gated Treg. Data are presented as mean±s.d.; each dot indicates one mouse (n=6 per group). ** $P < 0.01$, ns=not significant (two-tailed *t* test). Source data are provided as a Source Data file.

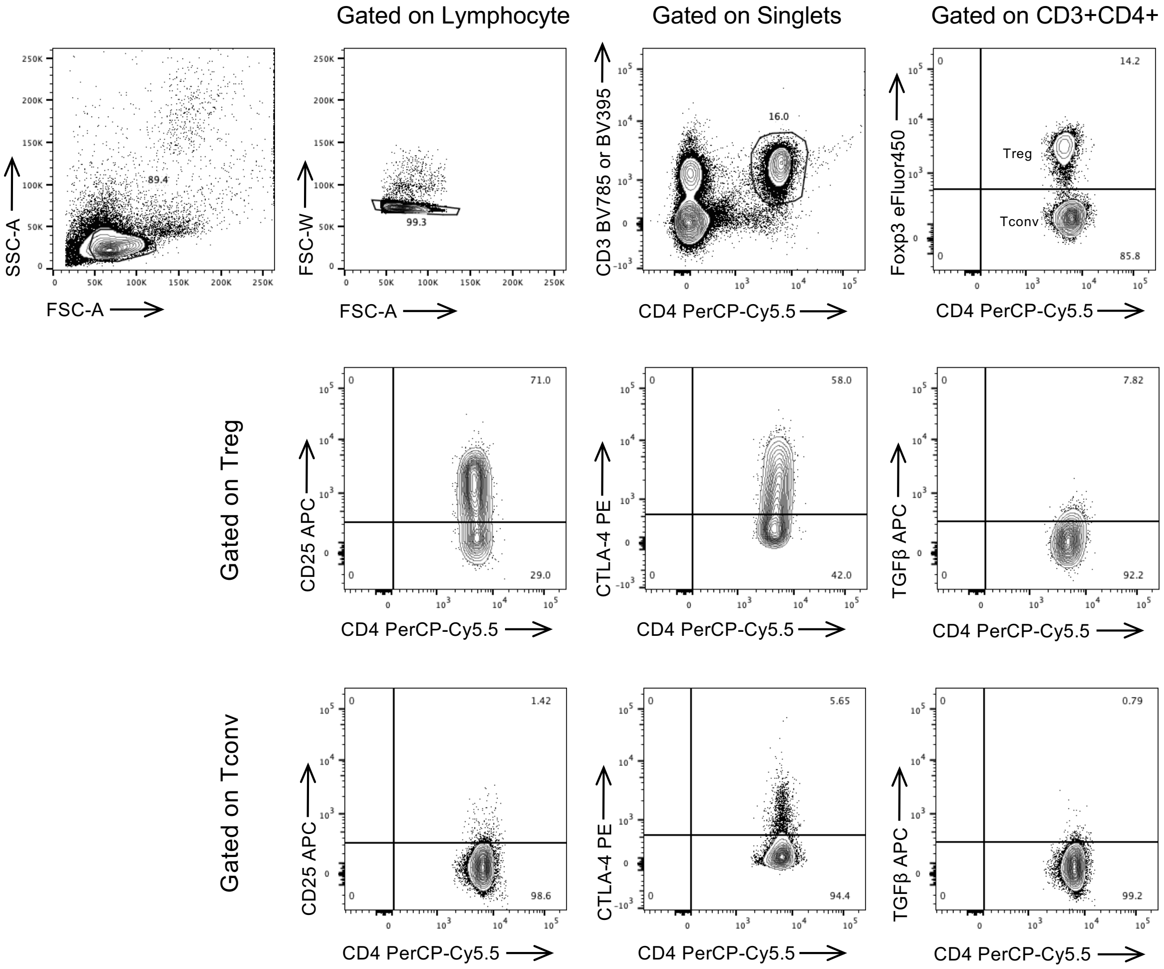

Supplementary Figure 9. Gating strategy for mice treated with short-term combination therapy in **Figure 3**. 7-15 week old BALB/c mice were injected i.p. with control Ab, anti-CD80/86 Ab, IL-2 complex or both anti-CD80/86 Ab and IL-2 complex as detailed in the methods. Spleen cells were analysed at d8. The FACS plots shown are from control Ab treated mice.
