# Supplementary material for: Costimulation blockade in combination with IL-2 permits regulatory T cell sparing immunomodulation that inhibits autoimmunity
Source: Nat Commun. 2022 Nov 9;13:6757. doi: 10.1038/s41467-022-34477-1 (PMC9643453; doi:10.1038/s41467-022-34477-1)
Supplement: Supplementary file 2 — Reporting Summary [file 41467_2022_34477_MOESM2_ESM.pdf]

nature portfolio

Corresponding author(s): Lucy S.K. Walker

Last updated by author(s): 16 Sep, 2022

Reporting Summary

Nature Portfolio wishes to improve the reproducibility of the work that we publish. This form provides structure for consistency and transparency in reporting. For further information on Nature Portfolio policies, see our Editorial Policies and the Editorial Policy Checklist.

Statistics

For all statistical analyses, confirm that the following items are present in the figure legend, table legend, main text, or Methods section.

n/a	Confirmed	
☐	☒	The exact sample size (n) for each experimental group/condition, given as a discrete number and unit of measurement
☐	☒	A statement on whether measurements were taken from distinct samples or whether the same sample was measured repeatedly
☐	☒	The statistical test(s) used AND whether they are one- or two-sided<br>*Only common tests should be described solely by name; describe more complex techniques in the Methods section.*
☒	☐	A description of all covariates tested
☐	☒	A description of any assumptions or corrections, such as tests of normality and adjustment for multiple comparisons
☐	☒	A full description of the statistical parameters including central tendency (e.g. means) or other basic estimates (e.g. regression coefficient) AND variation (e.g. standard deviation) or associated estimates of uncertainty (e.g. confidence intervals)
☐	☒	For null hypothesis testing, the test statistic (e.g. F, t, r) with confidence intervals, effect sizes, degrees of freedom and P value noted<br>*Give P values as exact values whenever suitable.*
☒	☐	For Bayesian analysis, information on the choice of priors and Markov chain Monte Carlo settings
☒	☐	For hierarchical and complex designs, identification of the appropriate level for tests and full reporting of outcomes
☒	☐	Estimates of effect sizes (e.g. Cohen's d, Pearson's r), indicating how they were calculated

Our web collection on statistics for biologists contains articles on many of the points above.

Software and code

Policy information about availability of computer code

Data collection	BD FACSDiva v8 or v9 (BD Biosciences), Summit 4.3 (Beckman Coulter), NDP.serve3, NIS elements AR 4.20.02
Data analysis	FlowJo v8 or v10 (BD Biosciences), Graphpad Prism v6 or v8, Qupath software v0.2.0-m4, Fiji (ImageJ) v2.0.0-rc-69/1.52p

For manuscripts utilizing custom algorithms or software that are central to the research but not yet described in published literature, software must be made available to editors and reviewers. We strongly encourage code deposition in a community repository (e.g. GitHub). See the Nature Portfolio guidelines for submitting code & software for further information.

Data

Policy information about availability of data

All manuscripts must include a data availability statement. This statement should provide the following information, where applicable:

- Accession codes, unique identifiers, or web links for publicly available datasets
- A description of any restrictions on data availability
- For clinical datasets or third party data, please ensure that the statement adheres to our policy

The data supporting the findings of this study are available within the article and its supplementary information files. Source data are provided with this paper.

Field-specific reporting

Life sciences study design

All studies must disclose on these points even when the disclosure is negative.

Sample size	There was no formal analysis to predetermine sample size. Minimum group sizes were determined for each set of experiments, using pilot data where available. For diabetes experiments, pilot data in the preventative setting (Fig. 4) indicated an effect size of 1.1370 (4 treatment groups; calculated using means & pooled standard deviation) and a total sample size of 16 (4 per group) (80% power, alpha of 0.05; GPower: https://pubmed.ncbi.nlm.nih.gov/17695343/). In practice, we used at least 6 per group in the light of the longer timeframe of the full experiments. Group sizes were larger in the therapeutic setting (Fig. 5) and in humanised mice experiments (Fig. 6) to allow for the greater inherent variability.
Data exclusions	Two mice were excluded from the humanized mice study because their circulating conventional CD4+ T cells had very low CD45RA staining (<5%). Two mice were excluded in Fig5b because they were culled early for reasons unrelated to the experiment. One mouse was excluded from Supplementary Fig8 because this mouse did not develop diabetes.
Replication	In vivo experiments were repeated at least three times and all attempts at replication were successful.
Randomization	For the majority of in vivo experiments, animals were randomly assigned into treatment groups after being matched for age or starting blood glucose.
Blinding	For in vivo experiments, mice were assigned numbers and numbers corresponding to specific group categories were not revealed until after analysis. For insulin and pancreas area quantification experiments, the treatment group was not revealed until after analysis.

Reporting for specific materials, systems and methods

We require information from authors about some types of materials, experimental systems and methods used in many studies. Here, indicate whether each material, system or method listed is relevant to your study. If you are not sure if a list item applies to your research, read the appropriate section before selecting a response.

Materials & experimental systems

n/a	Involved in the study	
☐	☒	Antibodies
☒	☐	Eukaryotic cell lines
☒	☐	Palaeontology and archaeology
☐	☒	Animals and other organisms
☒	☐	Human research participants
☒	☐	Clinical data
☒	☐	Dual use research of concern

Methods

n/a	Involved in the study	
☒	☐	ChIP-seq
☐	☒	Flow cytometry
☒	☐	MRI-based neuroimaging

Antibodies

Antibodies used

Target - Conjugate (Clone, Supplier, Catalogue No.)
Mouse CD4 PerCP (RM4-5, BD Biosciences, 553052)
Mouse Foxp3 eFluor 450 (FJK-16s, eBioscience, 48-5773-82)
Mouse Ki67 FITC (B56, BD Biosciences, 556026)
Mouse Foxp3 Pacific Blue (FJK-16s, eBioscience, 57-5773-80)
Mouse CD4 PerCP-Cy5.5 (RM4-5, BD Biosciences, 550954)
Mouse CD3 BV785 (17A2, BioLegend, 100232)
Mouse CD25 APC (PC61, BD Biosciences, 557192)
Mouse CTLA-4 PE (UC10-4F10-11, BD Biosciences, 553720)
Mouse CD3 BUV395 (145-2C11, BD Biosciences, 563565)
Mouse TGF-beta1 APC (TW7-16B4, BioLegend, 141405)
Mouse CD25 PE-Cy7 (PC61.5, eBioscience, 25-0251-82)
Mouse DO11.10 TCR PE (KJ126, eBioscience, 12-5808-82)
Mouse CTLA-4 PE-CF594 (UC10-4F10-11, BD Biosciences, 564332)
Mouse IL-2 functional grade (JES6-1A12, eBioscience, 16-7022-85)
Mouse CD4 BUV737 (RM4-5, BD Biosciences, 612843)
Mouse ICOS PE-Cy7 (7E.17G9, eBioscience, 25-9942-82)
Mouse CD103 BV786 (M290, BD Biosciences, 564322)
Mouse ST2 PerCP-eFluor710 (RMST2-2, eBioscience, 46-9335-82)
Mouse KLRG1 FITC (2F1, eBioscience, 11-5893-82)
Mouse CD39 Alexa Fluor 647 (Duha59, BioLegend, 143808)
Mouse CD73 PE (TY/11.8, BioLegend, 127206)
Human CD45 APC (HI30, eBioscience, 17-0459-42)

Human CD4 Alexa Fluor 700 (RPA-T4, BD Biosciences, 557922)
Human CD3 BUV395 (SK7, BD Biosciences, 564001)
Human Foxp3 PE (236A/E7, eBioscience, 12-4777-42)
Human CD25 BV421 (M-A251, BD Biosciences, 562442)
Human CTLA-4 BV786 (BNI3, BD Biosciences, 563931)
Human TGF-beta1 PE (TW4-2F8, BioLegend, 349604)
Human Foxp3 eFluor 450 (236A/E7, eBioscience, 48-4777-42)
Human CD45RA PerCP-Cy5.5 (HI100, eBioscience, 45-0458-42)
Human CD127 PE-Cy7 (ebioRDR5, eBioscience, 25-1278-42)
Mouse Purified rat anti-CD4 (GK1.5, BD Biosciences, 553727)
Mouse Foxp3 Alexa Fluor 488 (FJK-16s, eBioscience, 53-5773-82)
Mouse CD45 FITC (30-F11, eBioscience, 11-0451-85)
Mouse CD3 purified (145-2C11, BD Biosciences, 553058)
Rat Igs biotin (Polyclonal Sheep Igs, The Binding Site, AB331)
Mouse insulin unconjugated (Polyclonal Rabbit IgG, Santa Cruz, sc-9168)
Rabbit IgG (H+L) HRP (Polyclonal Goat IgG, Southern Biotech, 4050-05)
Mouse insulin unconjugated (Polyclonal Guinea pig, Dako A0564)
Mouse Glucagon unconjugated (Rabbit monoclonal EP3070, abcam, ab92517)
Armenian hamster IgG (H+L) Cy5 (Polyclonal Goat IgG, Jackson ImmunoResearch, 125-175-160-JIR)
Guinea pig IgG (H+L) Alexa Fluor 555 (Polyclonal Goat IgG, Invitrogen, A-21435)
Alexa Fluor 488 (Polyclonal Rabbit IgG, Invitrogen, A-11094)
Rabbit IgG (H+L) Alexa Fluor 488 (Polyclonal Donkey IgG, Invitrogen, A-21206)
Fluorescein/Oregon Green Alexa Fluor 488 (Polyclonal Goat IgG, Invitrogen, A-11096)
Rabbit IgG (H+L) Alexa Fluor 647 (Polyclonal Donkey IgG, Invitrogen, A-31573)

Validation

All antibodies were validated by manufacturers, human antibodies were titrated using PBMCs from a healthy donor prior to establishment of staining protocols. Mouse antibodies were titrated using mouse splenocytes. Antibodies for histology and confocal staining were titrated using mouse spleen or pancreas sections. Additional information for species and application can be found using Resource Identification Portal RRID identifiers or website provided.
Mouse CD4 PerCP: RRID:AB_394587
Mouse Foxp3 eFluor 450: RRID:AB_1518812
Mouse Ki67 FITC: RRID:AB_396302
Mouse Foxp3 Pacific Blue-replaced by Foxp3 eFluor 450: RRID:AB_1518812
Mouse CD4 PerCP-Cy5.5: RRID:AB_393977
Mouse CD3 BV785: RRID:AB_2562554
Mouse CD25 APC: RRID:AB_398623
Mouse CTLA-4 PE: RRID:AB_395005
Mouse CD3 BUV395: RRID:AB_2738278
Mouse TGF-beta1 APC: RRID:AB_10896418
Mouse CD25 PE-Cy7: RRID:AB_469608
Mouse DO11.10 TCR PE: RRID:AB_465943
Mouse CTLA-4 PE-CF594: RRID:AB_2732917
Mouse IL-2 functional grade: RRID:AB_469207
Mouse CD4 BUV737: RRID:AB_2870165
Mouse ICOS PE-Cy7: RRID:AB_2573564
Mouse CD103 BV786: RRID:AB_2738744
Mouse ST2 PerCP-eFluor 710: RRID:AB_2573883
Mouse KLRG1 FITC: RRID:AB_1311265
Mouse CD39 Alexa Fluor 647: RRID:AB_2563978
Mouse CD73 PE: RRID:AB_2154094
Human CD45 APC: RRID:AB_10667894
Human CD4 Alexa Fluor 700: RRID:AB_396943
Human CD3 BUV395: RRID:AB_2744382
Human Foxp3 PE: RRID:AB_1944444
Human CD25 BV421: RRID:AB_11154578
Human CTLA-4 BV786: RRID:AB_2738491
Human TGF-beta1 PE: RRID:AB_10645520
Human Foxp3 eFluor 450: RRID:AB_1548676
Human CD45RA PerCP-Cy5.5: RRID:AB_10718536
Human CD127 PE-Cy7: RRID:AB_1659672
Mouse Purified rat anti-CD4: RRID:AB_395011
Mouse Foxp3 Alexa Fluor 488: RRID:AB_763537
Mouse CD45 FITC: RRID:AB_465051
Mouse CD3 purified: RRID:AB_394591
Rat Igs biotin (Polyclonal Sheep Igs, The Binding Site, AB331): https://www.yumpu.com/en/document/view/35233936/immunobiological-reagents-binding-site
Mouse insulin unconjugated (Polyclonal Rabbit IgG): RRID:AB_2126540
Rabbit IgG (H+L) HRP (Polyclonal Goat IgG): RRID:AB_2795955
Mouse insulin unconjugated (Polyclonal Guinea pig): https://www.labome.com/product/Dako/A0564.html

Mouse Glucagon unconjugated (Rabbit monoclonal): RRID:AB_10561971
Armenian hamster IgG (H+L) Cy5 (Polyclonal Goat IgG): now replaced by Armenian hamster IgG (H+L) Alexa Fluor647 (Polyclonal Goat IgG): RRID: AB_2339001
Guinea pig IgG (H+L) Alexa Fluor 555 (Polyclonal Goat IgG): RRID:AB_2535856
Alexa Fluor 488 (Polyclonal Rabbit IgG): RRID:AB_221544
Rabbit IgG (H+L) Alexa Fluor 488 (Polyclonal Donkey IgG): RRID:AB_2535792
Fluorescein/Oregon Green Alexa Fluor 488 (Polyclonal Goat IgG): RRID:AB_221558
Rabbit IgG (H+L) Alexa Fluor 647 (Polyclonal Donkey IgG): RRID:AB_2536183

Animals and other organisms

Policy information about studies involving animals; ARRIVE guidelines recommended for reporting animal research

Laboratory animals	Species - Strain - Sex - age (when starting experiments)<br>Mouse - BALB/c - m and f - 6-15 week<br>Mouse - CD28-/- - m and f -6-8 week<br>Mouse - DO11 x RIPmOVA - m and f - 4-13 week<br>Mouse - NSG - m and f - 4-6 week
Wild animals	The study did not involve wild animals.
Field-collected samples	The study did not involve samples collected from the field.
Ethics oversight	Experiments were performed in accordance with the relevant Home Office regulations following institutional ethical approval (University of Birmingham Animal Welfare Ethical Review Body, University College London Animal Welfare Ethical Review Body).

Note that full information on the approval of the study protocol must also be provided in the manuscript.

Flow Cytometry

Plots

Confirm that:

- [x] The axis labels state the marker and fluorochrome used (e.g. CD4-FITC).
- [x] The axis scales are clearly visible. Include numbers along axes only for bottom left plot of group (a 'group' is an analysis of identical markers).
- [x] All plots are contour plots with outliers or pseudocolor plots.
- [x] A numerical value for number of cells or percentage (with statistics) is provided.

Methodology

Sample preparation	Mouse peripheral (inguinal, axillary and brachial) lymph node and pancreatic lymph node and spleen cells were obtained by crushing tissue through a wire mesh (Sigma-Aldrich). Red blood cells were removed from spleen and tail blood samples using lysis buffer. Lymphocytes were recovered from the pancreas by tearing the tissue into small pieces in cold PBS containing 5% FBS, 56 mM glucose (Sigma-Aldrich), 2 ug/ml Aprotinin (Roche) and 50 ug/ml TLCK (Roche). Following centrifugation, tissue was digested in pre-warmed PBS containing 304 ug/ml Liberase TL (Roche) and 10 ug/ml DNase (Sigma-Aldrich) at 37 degree for 12min in a shaking incubator. The reaction was then quenched with cold RPMI containing 10% FBS. Following centrifugation, the sample was resuspended in warm RPMI containing 10% FBS, passed through a 40 um cell strainer and layered onto Lympholyte-M (Tebu-bio). Lymphocytes were subsequently collected after centrifugation at 1000 g, at room temperature for 20min without brake, and washed in PBS containing 2% FBS.<br>Human CD34+ cells were isolated from cord blood using CD34 Microbead Kit UltraPure (Miltenyi Biotec).
Instrument	BD LSRFortessa (BD Bioscience), Dako CyAn ADP (Beckman Coulter)
Software	BD FACSDiva v8 or v9 (BD Biosciences) and Summit 4.3 (Beckman Coulter) were used for data collection. FlowJo software (v8 or v10) were used for data analysis
Cell population abundance	CD34+ cells purity > 90%
Gating strategy	CD28 blockade in vivo and IL-2 complex in vivo experiments:<br>forward scatter lin vs side scatter lin > forward scatter lin vs pulse width > gated on CD4+ > gated on CD4+Foxp3+, CD4+Foxp3- > CD4 vs Ki67<br>forward scatter area vs side scatter area > forward scatter area vs forward scatter width > gated on CD4+ > gated on CD4+Foxp3+, CD4+Foxp3- > CD4 vs Ki67<br>Short-term combination treatment (BALB/c):<br>forward scatter area vs side scatter area > forward scatter area vs forward scatter width > gated on CD4+CD3+ > Foxp3 vs CD25, Foxp3 vs CTLA-4, Foxp3 vs TGFβ<br>forward scatter area vs side scatter area > forward scatter area vs forward scatter width > gated on CD4+CD3+ > gated on CD4+Foxp3+, CD4+Foxp3- > CD4 vs CD25, CD4 vs CTLA-4, CD4 vs TGFβ<br>Short-term combination treatment (DO11 x RIPmOVA):

forward scatter lin vs side scatter lin > forward scatter lin vs pulse width > gated on CD4+ > gated on CD4+Foxp3+, CD4 +Foxp3-
forward scatter area vs side scatter area > forward scatter area vs forward scatter width > gated on CD4+ > gated on CD4 +Foxp3+, CD4+Foxp3-
Combination therapy in preventative setting experiments:
forward scatter area vs side scatter area > forward scatter area vs forward scatter width > gated on CD4+CD3+ > CD4 v DO11.10 TCR > CD4 vs Foxp3, CD25 v Foxp3
forward scatter area vs side scatter area > forward scatter area vs forward scatter width > gated on CD4+CD3+ > CD4 v DO11.10 TCR > CD4 vs Foxp3 > CD4 v CD25, CD4 v CTLA-4, CD4 v CD62L
Combination therapy in therapeutic setting experiments:
forward scatter area vs side scatter area > forward scatter area vs forward scatter width > gated on CD4+CD3+ > CD4 v DO11.10 TCR > CD4 vs Foxp3
forward scatter area vs side scatter area > forward scatter area vs forward scatter width > gated on CD4+CD3+ > CD4 vs Foxp3 > CD4 v DO11.10 TCR > CD4 v CD25, CD4 v CTLA-4
Combination therapy in humanize mice:
forward scatter area vs side scatter area > forward scatter area vs forward scatter width > gated on hCD45 > gated on hCD4 +hCD3+ > hCD4 v hFoxp3, hFoxp3 v hCD25, hFoxp3 v hCTLA-4, hFoxp3 v hTGFβ, hFoxp3 v hCD127
forward scatter area vs side scatter area > forward scatter area vs forward scatter width > gated on hCD45 > gated on hCD4 +hCD3+ > gated on CD4+Foxp3+ > hCD4 v hCD25, hCD4 v hCTLA-4, hCD4 v hTGFβ

☒ Tick this box to confirm that a figure exemplifying the gating strategy is provided in the Supplementary Information.
